# Single-cell imaging of protein dynamics of paralogs reveals sources of gene retention

**DOI:** 10.1016/j.isci.2025.112771

**Published:** 2025-05-27

**Authors:** Rohan Dandage, Mikhail Papkov, Brittany M. Greco, Vanessa Pereira, Dmytro Fishman, Helena Friesen, Kyle Wang, Erin B. Styles, Oren Kraus, Benjamin Grys, Gerardo Zapata, Francois Lefebvre, Guillaume Bourque, Charles Boone, Brenda J. Andrews, Leopold Parts, Elena Kuzmin

**Affiliations:** 1 Department of Biology, Concordia University, Montreal, QC, Canada; 2 Centre for Applied Synthetic Biology, Centre for Structural and Functional Genomics, Concordia University, Montreal, QC, Canada; 3 Institute of Computer Science, University of Tartu, Tartu, Estonia; 4 The Donnelly Centre, University of Toronto, Toronto, ON, Canada; 5 Department of Molecular Genetics, University of Toronto, Toronto, ON, Canada; 6 Department of Electrical and Computer Engineering, University of Toronto, Toronto, ON, Canada; 7 Canadian Centre for Computational Genomics (C3G), McGill University, Montreal, QC, Canada; 8 Department of Human Genetics, McGill University, Montreal, QC, Canada; 9 Victor Phillip Dahdaleh Institute of Genomic Medicine, Montreal, QC, Canada; 10 Wellcome Sanger Institute, Wellcome Genome Campus, Hinxton, Cambridgeshire, UK; 11 Rosalind & Morris Goodman Cancer Institute, McGill University, Montreal, QC, Canada

**Keywords:** Functional aspects of cell biology, Molecular biology, Phylogenetics

## Abstract

Gene duplication is common across the Tree of Life and contributes to genomic robustness. In this study, we examined changes in the subcellular localization and abundance of proteins in response to the deletion of their paralogs originating from the whole-genome duplication event, which is a largely unexplored mechanism of functional divergence. We performed a systematic single-cell imaging analysis of protein dynamics and screened subcellular redistribution of proteins. We find that 20% of proteins exhibit redistribution, of which 1/3 relocalized and 1/2 changed in abundance. Paralogs showed dependency, whereby proteins required their paralog to maintain their endogenous abundance or localization, 2-fold more often than compensation. Network feature analysis suggested the importance of functional redundancy and rewiring of protein and genetic interactions underlying redistribution paralog response. Translation of alternate protein isoform emerged as a compensatory mechanism. This study provides insight into paralog retention and evolutionary forces that shape genomes.

## Highlights

• Proteins change abundance or localization due to paralog loss
• Guided by rewiring and protein isoforms

## Graphical abstract

**Figure:**
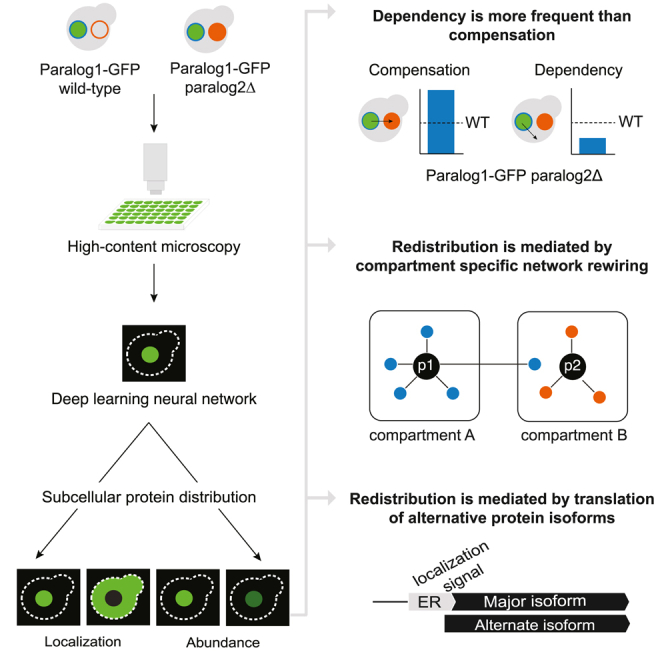

## Introduction

Gene duplication events play an important role in genome evolution and the emergence of adaptive complexity at the phenotypic level. Gene duplication occurs by polyploidy events resulting in whole genome duplicates (WGDs) or duplication of small genomic regions generating small-scale duplicates.^1^ Autopolyploidization, which is an intraspecies event occurring due to cytokinesis failure, or allopolyploidization, which results from an interspecies cross, are mechanisms that generate WGD.^1^^,^^2^^,^^3^ Small-scale duplications are thought to result from a variety of mechanisms, such as the presence of repetitive sequences that lead to unequal crossing-over, non-homologous recombination due to replication accidents, and horizontal gene transfer between organisms or transpositions.^4^^,^^5^ During the divergence of paralogs, a large fraction of them is removed due to nonfunctionalization.^6^ However, a significant proportion of the paralogs is retained, and this retention is thought to occur through three dominant routes^1^: (1) dosage amplification^7^ or backup compensation,^8^ (2) subfunctionalization, i.e., the partitioning of functions between the sister paralogs,^6^ and (3) neofunctionalization, i.e., the acquisition of novel functions.^9^

Systematic single gene deletion and loss-of-function screens in model organisms, such as yeast,^10^ worms,^11^ plants,^12^ and human cells^13^^,^^14^ have revealed that paralogs can provide genetic robustness. Consequently, paralogs tend to have a lower fitness cost when perturbed in these screens when compared to the perturbation of singleton genes. Genetic interactions have been used to study paralog divergence. Genetic interactions occur when a combination of mutations in different genes results in an unexpected phenotype, deviating from a model based on the integration of the individual mutant phenotypes.^15^ In yeast ∼30% of paralogs exhibit negative genetic interactions with each other, whereby the combined deletion of both paralogs results in a greater fitness defect compared to the effect of individual paralog deletions.^16^ This finding indicates the pervasiveness of the compensatory functional redundancy between paralogs. Complex genetic interaction screens involving double mutants of dispensable WGD paralogs in yeast also revealed that functionally redundant paralogs tend to have a relatively large fraction of compensatory negative trigenic interactions.^17^ According to the structural and functional entanglement model of duplicate divergence, the functional redundancy of paralogs is evolutionarily stable, as the divergence of functional domains is structurally constrained.^17^ Thus, genetic interaction studies have uncovered the prevalence of functional redundancy among retained paralogs.

The mechanisms leading to paralog retention involving protein and RNA dynamics are not completely understood. Previous studies have uncovered evidence consistent with a “responsive backup circuit” model where inactivation of a gene leads to the upregulation of a redundant paralog through changes to protein or mRNA expression levels.^18^^,^^19^^,^^20^ In these studies, the transcriptional upregulation of the gene or increased abundance of the protein revealed compensatory change in response to the loss of its paralog. Other studies showed that the inactivation of a gene leads to gain and/or loss of protein-protein (PPIs)^21^ and protein-DNA interactions.^22^ In these studies, the gain in interactions indicates compensation, whereas the loss of interactions indicates that the protein is dependent on the inactivated paralog. In the latter scenario, the paralogs effectively act as a single functional unit and thus, in contrast to compensation model, inactivating a dependent paralog can lead to a fitness defect as severe as inactivation of a singleton gene. Such dependency is common among the heteromeric paralogs that physically interact with each other, as reported in yeast^21^ and human cells.^14^

While changes in mRNA expression, protein abundance and protein interactions have been previously investigated as mechanisms of paralog retention, changes to protein localization have remained elusive despite evidence for subcellular localization differences among duplicated genes.^23^ The only known example of paralog compensatory localization changes comes from a previous study,^24^ which investigated the localizations of Stv1 and Vph1 in the budding yeast. This paralog pair encodes proteins belonging to the V-ATPase protein complex, which carries out the acidification of organelles.^25^^,^^26^ These paralogs localize to distinct subcellular compartments: Stv1 resides in the Golgi and endosomes, whereas Vph1 is found in the vacuole. The growth defect caused by the *VPH1* deletion was rescued by the overexpression of *STV1*, and the protein it encodes relocalized to the vacuole suggesting compensation for the loss of *VPH1*. This example suggests that largely under-investigated relocalization-based protein dynamics of paralogs may play a fundamental role in compensation, and thereby in retention of paralogs.

In yeast, previous high-throughput microscopy studies^27^^,^^28^^,^^29^^,^^30^ reported relocalization of the proteome in response to environmental, chemical or genetic perturbations and cataloged these response patterns.^31^ However, a systematic study of proteins through assessing their response to the loss of paralogs has been lacking. Such an investigation, afforded by the recent improvements in high-throughput microscopy techniques and quantitative analytic approaches, could answer fundamental questions related to paralog retention, such as: (1) at what prevalence proteins exhibit relocalization upon the deletion of their paralog, (2) how often the relocalizations are compensatory or dependent, (3) which properties of paralogs are predictive of relocalization, and (4) what mechanisms drive protein relocalization.

In this study, we used a phenomics approach to capture subcellular distribution changes of proteins in response to their paralog deletions and identify the functional relationship of paralogs. We compared GFP-tagged strains of proteins to mutant strains harboring deletions of their paralogs, identified their subcellular relocalizations and quantified protein abundance changes. The correlative analyses of evolutionary and physiological gene features revealed that the subcellular distribution changes of proteins are associated with paralog functional redundancy. We uncover alternate protein isoform abundance, which refers to protein variants of the canonical coding sequence, as a mechanism for paralog compensation.

## Results

### Quantifying paralog subcellular distributions using single-cell imaging

**Figure 1:**
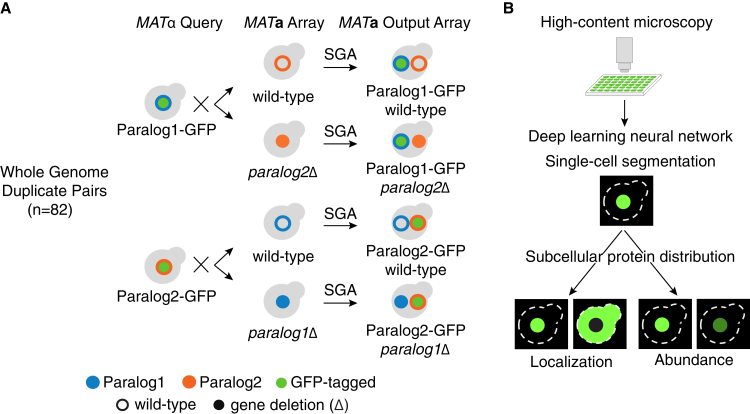
High-content protein dynamics analysis of paralogs (A) An illustration of strain construction for high-content screening of 82 paralog pairs originating from whole genome duplication (WGD) in yeast with previously reported localization differences. *MAT*α query strains harboring GFP-tagged proteins (green circle) were crossed with an array of *MAT*a strains harboring either wild-type (open blue or orange circles) or deletion (Δ) (solid blue or orange circles) of their paralog. After induction of meiosis in heterozygous double mutants, sequential replica-pinning steps of the synthetic genetic array (SGA) method were used to select the desired progeny, resulting in an output array of *MAT*a strains harboring GFP-tagged proteins in wild-type and deletion backgrounds of their paralogs. (B) High-content screening and analysis of the array of strains harboring GFP-tagged proteins in wild type and deletion backgrounds of their respective paralogs. The strains were imaged using automated high-content microscopy. A deep neural network was used for cell segmentation and feature extraction to capture the subcellular protein distribution, which is a collective measure of protein abundance and localization.

We constructed 328 yeast strains, enabling us to interrogate 82 WGD paralog pairs, each of which harbored a GFP-tagged protein in a wild-type or deletion of its paralog. These paralogs encoded proteins that exhibit distinct subcellular localizations^28^^,^^29^^,^^32^ and represent 82 of 551 of unique WGD paralog pairs.^33^ We used the synthetic genetic array (SGA) approach to generate four strains per paralog pair by crossing strains carrying GFP-tagged proteins with strains carrying a wild-type or deletion of their paralog (Figure 1A, STAR Methods). These strains enabled the assessment of the subcellular protein distribution changes in a reciprocal manner. Six proteins were paired with random paralogs to serve as negative controls and a proxy for the singletons, because of their significantly weaker sequence alignments compared to the paralog pairs (Figure S1). Use of such controls overcomes the challenge of additional strain construction, phenomic screening and quantification and sampling the expansive space of singleton pairs.

Using high-content microscopy, we measured changes to the subcellular localization and abundance of each protein in response to the deletion of its paralog (Figure 1B). We used an automated image analysis pipeline involving a deep neural network for single-cell segmentation and feature extraction (Figure S2). This amounted to a total of ∼3,500 images and ∼460,000 single cells, which necessitated the use of an automated machine learning approach for detecting protein dynamics, as done previously in high-throughput phenomics studies.^30^^,^^31^ This approach also overcomes the inherent limitations of the visual inspection for a quantitative image analysis. With the automated approach, ResNet-18 neural network architecture was used to extract 128 features for each single cell, which were used to capture protein distributions, including protein localization and protein abundance (Data S1). Images with artifacts or low number of cells were excluded from the analysis. Visual inspection of the resulting images revealed wild-type subcellular localization of paralogs that was consistent with previous studies.^29^^,^^34^ We also obtained single-cell level protein abundances from the images by quantifying the mean pixel intensity per cell (Data S2). The resulting abundance scores in the wild-type background correlated well with previous measurements of protein abundance^35^ (Figure S3A) and showed a strong correlation across replicates (Figure S3B).

### Protein dynamics reveal scenarios of compensation and dependency

**Figure 2:**
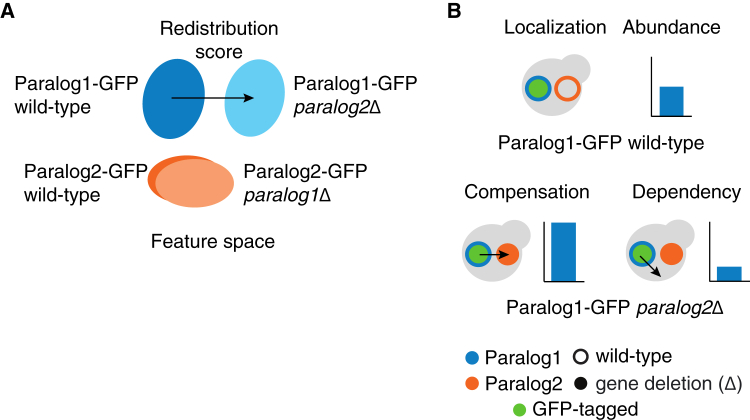
Identifying cases of compensation and dependency of paralogs using protein redistribution analysis (A) Schematic representation of the redistribution of the paralogs in the feature space (set of features extracted from deep neural network). The protein redistribution of a paralog between wild-type (dark blue/orange) and the deletion (light blue/orange) backgrounds is indicated by the arrow. (B) Scenarios of the compensation and dependency for relocalization and relative abundance change. An example is depicted in which GFP-tagged protein 1 (green circle with blue border) is in the wild-type (open orange circle) or deletion (Δ) (solid orange circles) background of its paralog. Compensation refers to an increased protein abundance or relocalization to its paralogous protein subcellular compartment in response to the deletion of its paralog (left), whereas dependency refers to a decreased protein abundance or relocalization to a subcellular compartment distinct from its own or of its paralogous protein in response to the deletion of the paralog (right). Arrow denotes localization change.

To capture changes in subcellular localization and abundance of proteins in response to their paralog deletion, we calculated the changes in their protein distribution in the presence or absence of their paralog. We defined a redistribution score as the Euclidean distance between the centroid points of the extracted features comparing backgrounds for each protein (Figure 2A, STAR Methods). The redistribution score is a collective measure of both the relative change in protein abundance and localization. We also separately quantified relative protein abundance changes for these paralogs.

The protein dynamics between paralogs should be captured by the response of proteins they encode to the deletion of their paralog when assessing their subcellular localization and abundance. Using the redistribution scores and relative protein abundance changes, we assessed the protein dynamics of paralogs. We predict that compensation would be observed when proteins respond to the loss of their paralog by changing their subcellular localization to reside in their paralog’s compartment and/or increasing their protein abundance. In contrast, dependency would be observed when proteins respond by diverging from their own or their paralogous protein’s wild-type subcellular localization and/or decreasing in abundance (Figure 2B).

### Proteins respond to their paralog loss by subcellular redistribution

**Figure 3:**
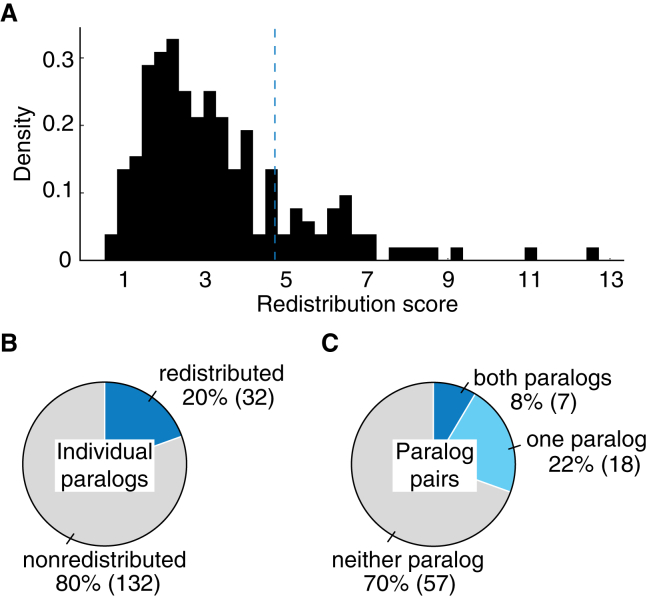
Proteins respond to the loss of their paralog by protein redistribution (A) Histogram of the redistribution scores of screened paralogs. Redistribution score of greater than 4.73 (dashed line) was used to classify paralogs that exhibit protein redistribution. (B) Pie chart showing the number of proteins that exhibited redistribution in response to their paralog deletion (blue). Paralogs that show no response are depicted in gray. (C) Pie chart showing the number of paralogous protein pairs that exhibited redistribution in response to their paralog deletion. Pairs in which both paralogs responded to each other’s deletion are depicted in blue, those in which one paralog responded are in light blue, paralog that did not respond are in gray.

In total, we quantified the redistribution scores for 164 proteins comprising 82 paralog pairs. These proteins showed a range of redistribution scores (Figure 3A), with 132 paralogs exhibiting a relatively low redistribution score (below 4.73), while a smaller subset of 32 paralogs belonging to 25 paralog pairs displayed a higher redistribution score (Figure 3B). The scoring was consistent with independent visual inspection, indicating that paralogs with relatively high redistribution scores exhibited subcellular localization and/or protein abundance changes, and randomly paired paralogs showed low redistribution scores (Table S3). Also expectedly, the non-redistributed paralogs showed no change in protein dynamics, as validated through visual inspection.

Based on the randomized controls and visual inspection, we next obtained a threshold to classify the redistributed and non-redistributed paralogs (Figure S4A). In this classification, we considered the individual paralogs instead of pairs, to avoid potentially confounding interference of asymmetric effects within paralog pairs. At this threshold, the ∼17% false negative rate observed for controls indicates a high sensitivity when classifying paralogs. A low fraction of redistributed paralogs, showing borderline redistribution scores close to the threshold were classified as non-redistributed. On the other hand, the false positive rate observed for controls entails that when the optimized threshold is applied to the paralogs, the false positives are expected to be minimal, therefore optimizing specificity. However, this is an estimate and false positives may still be present in the dataset due to the high-throughput nature of this study. This stringent threshold prioritizes the removal of false positives, which is crucial in the analysis of large-scale single-cell data. Paralog pairs having only one responsive member (18 pairs) were more common than reciprocal responsiveness, where both proteins respond to each other’s deletion (7 pairs, Figure 3C). This finding is consistent with the asymmetric divergence of paralogs.^16^^,^^36^

### Proteins respond to paralog loss by abundance change

**Figure 4:**
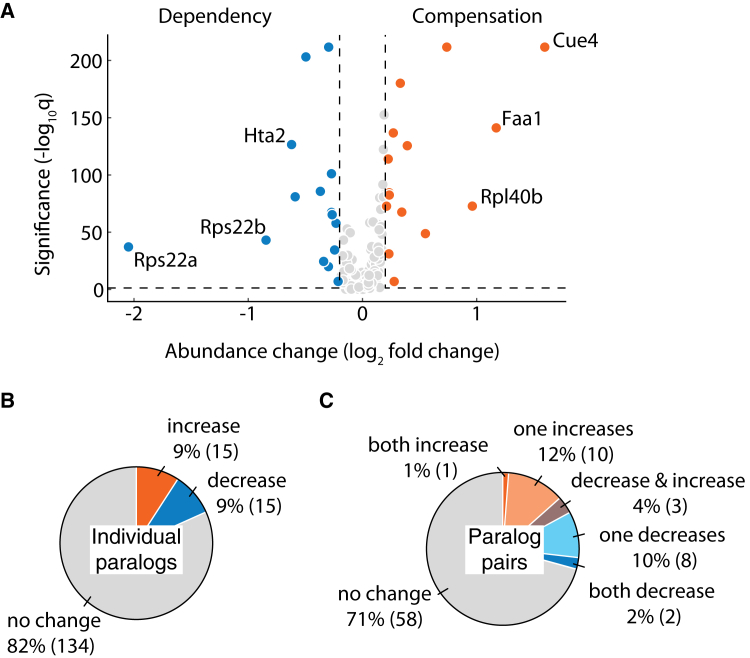
Proteins respond to their paralog loss by protein abundance changes (A) Volcano plot showing the protein abundance changes. The paralogs exhibiting significant relative abundance increase (log_2_ fold change ≥0.2, *q* < 0.05), suggesting compensation, are shown in orange, whereas the ones exhibiting decrease (log_2_ fold change ≤ −0.2, *q* < 0.05), suggesting dependency, are shown in blue. The labeled points correspond to the top 3 proteins showing the greatest compensation and dependency. The points in gray correspond to the proteins that show nonsignificant changes. *q*: FDR-corrected *p* value. (B) Pie chart showing the number of individual proteins that exhibited significant protein abundance increase (orange), decrease (blue) or no significant change (gray) in response to their paralog deletion. (C) Pie chart showing the number of paralog pairs that exhibited protein abundance change in response to their paralog deletion. Paralog pairs in which both proteins show increased abundance are shown in orange and those in which one protein increased are in light orange. Paralog pairs in which both proteins show decreased abundance are shown in blue and those in which one protein decreased are in light blue. Violet color depicts the paralog pairs in which proteins responded in opposite directions. Paralog pairs that showed no response are shown in gray.

To test whether the redistribution score captures protein abundance changes, we separately quantified the abundance of each protein in the presence and deletion of its paralog (Figure 4A). We identified 30 proteins that exhibited a significant change in abundance upon the deletion of its paralog (|log_2_ fold change| ≥ 0.2, *p* < 0.05) (STAR Methods). The relative protein abundance change is consistent with a previous study.^19^ For example, Cue4, a protein of unknown function, and Pgm2, a phosphoglucomutase, which is involved in hexose metabolism,^37^ were upregulated, whereas Sds24, a protein involved in cell separation during budding,^38^ was downregulated in both studies. Despite the differences in the methods used, the similarity in the classification supports the reproducibility of the relative abundance changes measured in the study. In total, 16 out of 30 proteins that showed significant relative abundance changes also exhibited redistribution. The remaining 14 proteins, which were not classified as redistributed tended to show a modest protein abundance change, and thus not being detected by the redistribution score representing false negatives (Figure S4B). Overall, there was an equal number of paralogs showing a relative increase in protein abundance, suggesting compensation, as there were paralogs showing a relative decrease in protein abundance, suggesting dependency (Figure 4B).

We also observed that compensation and dependency can co-occur in the same protein complex. Rps22a-Rps22b belong to the small ribosomal subunit and both show decreased abundance in response to each other’s loss indicating reciprocal dependency (Figures 4A, S5A–S5C). On the other hand, Rpl40a-Rpl40b belong to the large ribosomal subunit with Rpl40b increasing in abundance due to the loss of its paralog suggesting compensation (Figures 4A, S5D–S5F). Similar to redistribution (Figure 3C), the relative abundance changes in one member of the paralog pair were more common than reciprocal changes in both paralogs (Figure 4C). This observation was not confounded by the asymmetry in the wild-type abundances of the paralogs. There was no significant association between the relative abundance changes and (1) the asymmetry (*p* = 1, Mann-Whitney U test) or (2) the under-expression of one of the paralogs (*p* = 0.6, Mann-Whitney U test) in the wild-type background. Therefore, the asymmetric relative abundance changes reflect the property of protein response to their paralog loss.

### Subcellular relocalization of paralogs reveal cases of compensation and dependency

**Figure 5:**
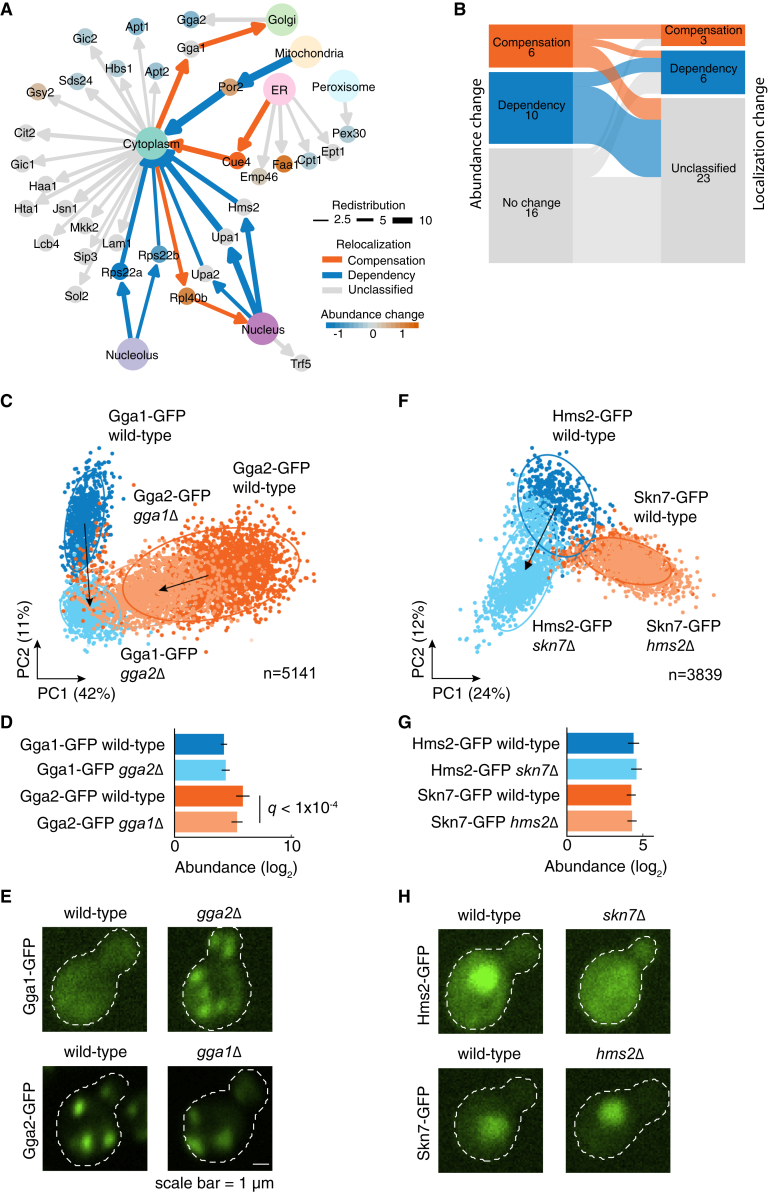
Proteins redistribute in response to their paralog loss by abundance and localization changes (A) Summary network of paralog redistribution. Redistribution score (edge width), abundance change (node color), protein relocalization (edges directed to subcellular compartment). Edges show relocalization and nodes show abundance change. The compensation and dependency are shown by blue and orange colors, respectively. (B) Comparison of proteins showing compensation (blue) and dependency (orange) between the relative abundance change and relocalization. (C) Redistribution of the Gga1-Gga2 pair is visually represented on a dimensionality reduced principal-component analysis (PCA) space of *Z* score normalized features for each protein. Redistributed paralogs are shown with the arrow connecting the centroids of the clusters corresponding to the wild-type (dark blue/orange) and deletion (light blue/orange) backgrounds. The percentage variances explained by the PCs are indicated in parentheses. The PCA plot is used for visualization of the redistribution only. (D) Relative abundance changes of the Gga1-Gga2 pair. The error bars show the 95% confidence intervals of the means. *q*: FDR-corrected *p* value. Gga1-GFP is shown in blue and Gga2-GFP in orange. Wild-type paralog background is shown in dark blue/orange and deletion paralog background in light blue/orange backgrounds. (E) Micrographs of representative yeast cells showing compensatory relocalization of Gga1-GFP in response to *GGA2* deletion and abundance dependency of Gga2-GFP in response to *GGA1* deletion. (F–H) Similar to panels as C–E, but for the Hms2-Skn7 pair. The redistribution, relative abundance change, and micrographs are shown. The micrographs in panel H show the dependent relocalization of Hms2-GFP in response to *SKN7* deletion.

To further explore paralog redistribution, we considered subcellular localization changes. In total, among 32 proteins that showed redistribution in response to their paralog deletion, we used visual inspection and detected relocalization in 9 proteins (Cue4, Gga1, Hms2, Por2, Rpl40b, Rps22a, Rps22b, Upa1, and Upa2) from 7 paralog pairs (Figures 5A, S5, Table S4). Reciprocal cases in which both proteins relocalize are rare (2 of 7, Rps22a-Rps22b, and Upa1-Upa2), similar to the rarity of reciprocal protein abundance changes. Relocalization was accompanied by protein abundance changes for 5 out of 9 proteins, indicating that redistribution captures both changes. The remaining 12 out of 32 proteins that were scored as redistributed were neither classified as relocalized nor showed relative abundance change. These paralogs may exhibit relocalization too subtle to be confidently detected by visual inspection but may still represent real biological response and follow-up experiments should be designed to better understand their response and differentiate them from false positives. As expected, the randomly paired paralogs, serving as negative controls, exhibited neither abundance change nor relocalization.

Genetic backgrounds have been shown to influence cellular morphology.^39^ The deep learning model we have used to obtain features of protein dynamics and then to calculate the redistribution scores, by design, may look at shapes and sizes, but is explicitly trained to ignore this signal. Nonetheless, we tested whether the redistribution scores are robust to the morphological changes (Figure S6A). We find that the neural network derived 128 features that are used to calculate redistribution scores are weakly correlated with morphological characteristics, whereby most of these correlations are not statistically significant (Figure S6B). For the few that are significant, most of them are for the correlations with the paralogs that exhibited abundance change (Figure S6C). Thus, since the correlations with morphological characteristics themselves are low, they exert a weak confounding effect on redistribution scores that we attribute to protein abundance change.

Since the redistribution score is not directional, we considered the nature of the abundance and subcellular localization changes to identify cases of compensation and dependency. Considering proteins that respond to the deletion of their paralog by subcellular relocalization, we categorized three proteins as exhibiting compensation: Cue4, Gga1, and Rpl40b and six proteins as exhibiting dependency: Hms2, Por2, Rps22a, Rps22b, Upa1, and Upa2 (Figure 5A). We find that the compensation and dependency of relocalization and relative abundance changes often co-occur (Figure 5B). The redistributed proteins that show abundance compensation are more likely to show relocalization compensation, such as Cue4 and Rpl40b, except for Por2, which shows dependent relocalization. Similarly, redistributed proteins that show abundance dependency also exhibit relocalization dependency. Overall, among the redistributed paralogs, abundance and relocalization dependency was more prevalent than compensation.

Independent of the high-throughput screen, we re-imaged and validated two paralog pairs exhibiting redistribution, Gga1-Gga2, and Skn7-Hms2 (STAR Methods), which represent cases of relocalization compensation and dependency. Among them, the Gga1-Gga2 pair is involved in facilitation of Golgi trafficking.^40^ This pair is reciprocally redistributed, as visually represented on a dimensionality reduced space in which the feature space for Gga1-GFP and Gga2-GFP cells differs in the wild type and the deletion background of their respective paralogs (Figure 5C). This is due to Gga2 dependent relative abundance change (Figure 5D) and Gga1 compensatory relocalization from diffuse cytoplasm to pronounced Golgi punctae, observed for the wild-type localization of Gga2 (Figure 5E).

On the other hand, Skn7-Hms2 is a paralog pair that showed dependent localization. Skn7 is a transcription factor required for the optimal induction of heat-shock response to oxidative stress^41^ and osmoregulation.^42^ Hms2 is similar to heat shock transcription factors, although poorly characterized. It redistributes in response to *SKN7* deletion (Figure 5F), with no abundance change (Figure 5G), but relocalizes from the nucleus to the cytoplasm (Figure 5H). The cellular compartment where Hms2 relocalizes is different from its wild-type localization and that of its paralog, indicating a dependent relocalization. Overall, we found that the relocalization and the relative abundance changes of paralogs can collectively or individually manifest as their redistribution.

### Properties of paralogs are predictive of redistribution

**Figure 6:**
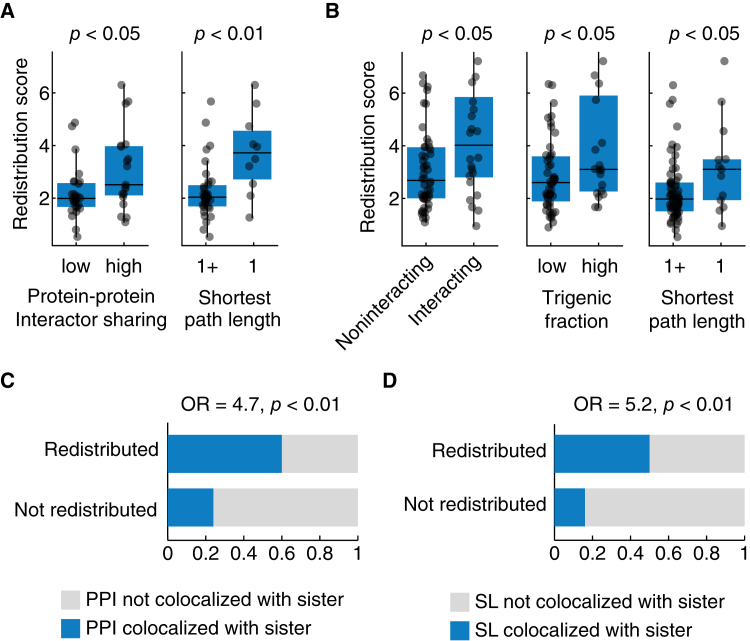
Redistribution correlates with protein-protein and genetic interaction network features of paralogs (A) Protein-protein interaction (PPI) network features. Plots show redistribution scores of individual paralogs, which were binned according to their shared PPI with their paralog (left) or the shortest path length between paralogs (right). Paralogs with fewer or more than the median number of shared interactors are denoted as low and high, respectively. The shortest path length of 1 (denoted as 1) and more than 1 (denoted as 1+) in the interaction network is shown. Merged PPI functional standard was used (STAR Methods). (B) Genetic interaction network features. Plots show redistribution scores of individual paralogs, which were binned according to the genetic interaction network features, including: (left) paralogs that exhibit a negative genetic interaction (interacting) or no interaction (noninteracting) within the paralog pair (previously defined stringent threshold i.e., *p* < 0.05, ε < −0.12)^43^; (middle) paralogs belonging to the previously defined high and low trigenic interaction fraction classes^17^; and (right) the shortest path length of 1 (denoted as 1) and more than 1 (denoted as 1+) in the genetic interaction network (stringent threshold i.e., *p* < 0.05 and ε > 0.16 or ε < −0.12).^43^ (C) Redistributed proteins show interactions in the subcellular compartment of their paralog. The association of the redistribution and private interactors’ colocalization with paralog, is shown for PPI and (D) the synthetic lethal genetic interactors (right) (*p* < 0.05 and ε < −0.35).^43^ OR: Odds ratio; *p*: *p* value obtained from Fisher’s exact test. In the boxplots, the central line indicates the median, the extent of the box is from the first quartile (Q1) to the third quartile (Q3), and the whiskers extend to Q1-1.5∗IQR and Q3+1.5∗IQR. Statistical significance was determined using a two-sided Mann-Whitney U-test.

After establishing the extent of redistribution and compensation, we next asked which paralog properties can predict these phenomena. Paralogs with a relatively high redistribution score are more likely to exhibit overlapping PPIs and have a shorter path length indicating that they are closely situated in the PPI network (Figure 6A). We find that paralogs with negative genetic interactions with each other were associated with a relatively high redistribution score (Figure 6B, left), indicating that the loss of both paralogs in a pair leads to a greater fitness defect compared to the fitness defect due to the loss of individual paralogs. A high redistribution score was also associated with a high trigenic fraction (Figure 6B, middle) due to a greater number of compensatory negative trigenic interactions compared to paralog-specific digenic interactions, indicating paralogs with a functional overlap. Also, similar to the PPI network, paralogs with a relatively high redistribution score were also closely located in the genetic interaction network (Figure 6B, right). We did not detect a bias in these features when comparing paralogs examined in this study with all the remaining WGD paralogs (Figures S7A and S7B), with the exception of negative genetic interactions, which were underrepresented in the latter, highlighting the predictive strength of these features for redistributed paralogs. Together these findings suggest that the functional redundancy may act as a prerequisite for paralogs to exhibit redistribution.

The subcellular redistribution of paralogs in response to the deletion of their sister paralogs may also be related to network rewiring. We found that compared to non-redistributed paralogous proteins, redistributed paralogous proteins are more likely to interact with other proteins residing in the subcellular compartment of their paralog (Figure 6C). This trend was also recapitulated using genetic interactions, whereby redistributed paralogs showed synthetic lethal interactions with proteins residing in the subcellular localization of their paralog (Figure 6D). We restricted this analysis to the private interactors, which are unique to each paralog, to eliminate the confounding effects of the shared interactors of the paralogs. We also did not detect a bias in these features, when comparing paralogs examined in this study with all the remaining WGD paralogs (Figures S7C and S7D), further highlighting the predictive strength of these features for redistributed paralogs. This finding suggests that possessing interactors in the paralog localization might be a prerequisite for network rewiring associated with redistribution.

To gain finer insights into how interactions may change due to paralog loss, we compared the redistribution of paralogs with previously reported PPI changes of paralogs.^21^ Consistent with our expectation, among 10 paralogs that overlapped both studies, 7 paralogs with a PPI change exhibited relatively high redistribution scores (Figure S8A). These findings suggest that PPI changes are predictive of redistribution. Thus, protein redistribution in response to their paralog loss occurs due to network rewiring which is likely mediated by compartment-specific PPIs.

### Compensatory relocalization and alternate protein isoform abundance

**Figure 7:**
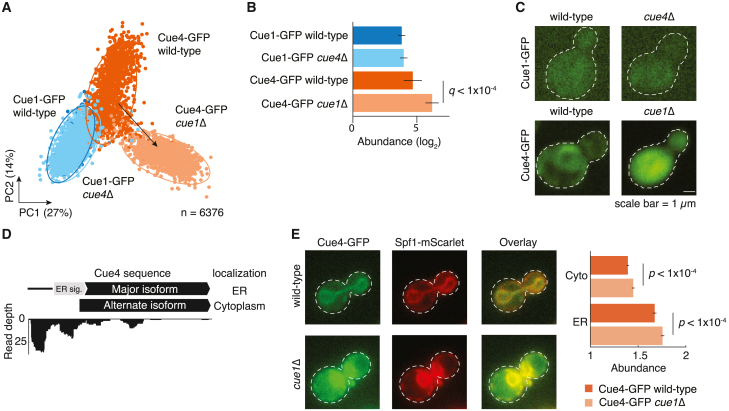
Redistribution of Cue4 and alternate protein isoform abundance (A) Redistribution of the Cue1-Cue4 pair is visually represented on a dimensionality reduced PCA score of *Z* score normalized features for each construct. Redistributed paralog is depicted by the arrow connecting the centroids of the clusters corresponding to the wild-type (dark blue/orange) and deletion (light blue/orange) backgrounds of the paralog. The percentage variances explained by the PCs are indicated in parentheses. The PCA plot is used for visualization of the redistribution only. (B) Relative abundance changes of the Cue1-Cue4 pair. The error bars show the 95% confidence intervals of the means. *q*: FDR-corrected *p* value. Cue1-GFP is shown in blue and Cue4-GFP is in orange. Wild-type background is shown in dark blue/orange and deletion background of the paralog in light blue/orange. (C) Micrographs of representative yeast cells from the high-throughput screen showing compensatory relocalization of Cue4-GFP in response to *CUE1* deletion. (D) The major and alternate protein isoforms of Cue4 (top) and corresponding read depths of ribosome footprints^46^ are shown. Predicted subcellular localizations of Cue4 isoforms are on the right. (E) Cytoplasmic and ER abundance of Cue4-GFP in response to *CUE1* deletion was monitored. Spf1-mScarlet was used as a reporter of ER. The error bars show the 95% confidence intervals of the means, *p* value calculated using Mann-Whitney U test.

To further explore the mechanisms underlying paralog redistribution, we examined in detail the *CUE1-CUE4* paralog pair. The pair encodes proteins containing a coupling ubiquitin conjugation to ER degradation (CUE) domain that binds to ubiquitin.^44^ Cue1 is involved in the ER-associated degradation (ERAD) pathway for misfolded proteins,^45^ whereas *CUE4* encodes a protein of unknown function. Cue1 is localized in the ER and cytoplasm, whereas Cue4 is predominantly localized in the ER.^32^^,^^34^ The Cue4 protein exhibited redistribution (Figure 7A), which was due to a compensatory relative abundance increase (Figures 7B, S8B) and compensatory relocalization to the cytoplasm (Figure 7C). This suggests an overall compensatory redistribution for the loss of *CUE1*.

Since Cue4 contains an N-terminal ER targeting signal sequence, we hypothesized that an N-terminal truncation might render the protein cytoplasmic. We mined the translation initiation site (TIS) profiling data^46^ and identified an alternate isoform of Cue4. This isoform lacks the N-terminal ER targeting signal sequence due N-terminal truncation caused by the translation from an alternative start site. It is therefore predicted to be localized to the cytoplasm (Figure 7D). This prediction is robust to GFP-tagging of the protein sequences (STAR Methods). A previously reported gain of an interaction between Cue4 and Ilm1, a poorly characterized cytoplasmic protein, in *cue1*Δ mutant background is consistent with the presence of an alternate cytoplasmic protein isoform of Cue4 (Figure S8C).^21^ Validation experiments in which we quantified Cue4 abundance in the ER and the cytoplasmic compartments showed that Cue4 protein abundance was greater in ER and cytoplasm in *cue1*Δ mutant compared to the wild-type background (Figure 7E). The Cue4 compensatory increase in abundance is consistent with the large-scale screen and the observed redistribution of Cue4 to the cytoplasm is due to increased translation of both protein isoforms.

## Discussion

Investigating protein dynamics underlying paralog retention has the potential to uncover how the fundamental process of genome evolution is fueled by gene duplication. In this study, we identified proteins that responded to the deletion of their WGD paralog by subcellular protein redistribution (Figure 3A), capturing relative abundance and localization changes (Figure 5A). We found that 20% of proteins redistributed in response to the deletion of their paralog, of which 28% showed relocalization, 50% showed relative abundance change, and 16% showed both relocalization and abundance change. The modest fraction of redistributed proteins is likely because of the known need-based responsiveness of paralogs, whereby the response is detected only in alternative environmental conditions,^19^ whereas our study was conducted in a nutrient-rich condition. The properties of paralogs that are predictive of redistribution include shared PPIs, short path length on the protein-protein and genetic interaction networks, negative genetic interactions within a paralog pair and high trigenic interaction fraction. Thus, protein-protein and genetic interaction network features revealed functional similarity as a predictor of paralog redistribution (Figures 6A and 6B). Functional similarity is considered a key characteristic of functional redundancy, which has been shown to be evolutionary stable.^17^^,^^47^ We also find that rewiring of PPIs in specific subcellular compartments may guide the relocalization of paralogs since we detected co-localization of protein interactions and synthetic lethal interactions of the disrupted protein with the redistributed paralog (Figures 6C and 6D). However, the sensitivity of the redistribution score may influence the strength of these correlations. Finally, we uncovered a compensatory mechanism resulting from paralog relocalization involving the translation of alternate protein isoform.

In our data, we observed that the paralogs from the small ribosomal subunit exhibited responses that were opposite to those of the paralog from the large subunit. This finding adds to the recent recognition that ribosomes are not homogeneous, but rather heterogeneous macromolecular complexes.^48^ Ribosomal duplicate genes generate heterogeneity in the compositions of the ribosomes and carry out moonlighting functions,^49^^,^^50^ which collectively contribute to the robustness and phenotypic plasticity.

Our finding that Hms2 shows a dependent localization change in response to *SKN7* deletion is consistent with this pair thought to form a heterodimer.^22^ The asymmetric nature of the dependency is likely due to homomerization of Skn7^51^ but not of Hms2, which enables Skn7 to function in the absence of Hms2 but not vice versa. This is also consistent with a previous study examining DNA binding preferences, which showed that Hms2 loses binding to its target genes in response to the deletion of *SKN7.*^22^

The analysis of alternate translation start sites of Cue4 is consistent with its compensatory relocalization occurring due to alternate protein isoform abundance. Similar to our findings in *cue1*Δ background, a previous study also reported that Cue4 relocalizes to the cytoplasm in the *rpd3*Δ background.^29^^,^^32^ Rpd3 is a lysine deacetylase with a diverse set of substrates,^52^ and thus it is possible that it shares common substrates with ubiquitin ligases that interact with Cue1 and impinge on Cue4 function. The similar effect of the loss of *RPD3* and *CUE1* on Cue4 may reflect their roles in maintaining proteostasis, albeit in distinct ways. Rpd3 is required for the inactivation of ribosomal genes^53^; therefore, its deletion may lead to dysregulation of translation and proteostatic stress. Because Cue1 is involved in protein degradation,^45^ its deletion may increase the load of undegraded proteins. Therefore, both *cue1*Δ and *rpd3*Δ might have a converging effect of increased proteostatic stress. Thus, Cue4 relocalization to the cytoplasm and its increased abundance could relieve the proteostatic stress by enabling additional protein degradation. Collectively, our study provides a molecular mechanism explaining subcellular protein dynamics of paralogs that underlie their retention within the yeast genome. The mechanisms of relocalization changes of paralogs were hypothesized^54^ but never systematically tested prior to this study.

We found variation in protein dynamics, as captured by protein relocalization and abundance changes. Reciprocal redistribution of paralogs was found to be rare. We speculate that the asymmetric nature of the divergence of the paralogs may be the fundamental reason for the asymmetry. In cases where reciprocal redistribution did occur, both paralogs responded by compensation or both by dependency. This finding was consistent with a previous study examining PPIs of paralogs^21^ and “monochromatic” genetic interactions involving members of pathways and protein complexes, indicating cellular organization into functional modules.^55^^,^^56^

In yeast, since WGD occurred around the time of the emergence of fruiting plants,^57^ changes to the metabolic milieu may have exerted selective pressures for paralog retention. To account for such effects, carrying out the screening under different environmental conditions would be necessary to reveal environment-specific paralog responses. Such screenings would also reveal which protein dynamics are robust to environmental changes and which are not.

Extending the approach of phenomic screenings as presented in this study to human cells, where paralogs are more prevalent than in yeast, could reveal unexplored mechanisms explaining paralog retention and the genetic robustness of human cells.^14^ Such an extension may also help in characterizing potential subcellular relocalizations of human paralogs due to alternate isoforms resulting from splicing. The coupling of functional redundancy of paralogs with splice forms, which can be dubbed as “internal-paralogs”,^58^ may be the key to explaining the exceptional genetic robustness of human cells. Furthermore, such fundamental understanding provide insight into targeted cancer interventions, where synthetic lethality-based precision oncology therapeutic strategies could be devised to inhibit compensatory paralogs by blocking their redistribution, thereby specifically killing tumor cells while leaving normal cells unharmed.

The models of paralog retention, which have been outlined through decades of research in genomics and functional genomics, have paved the way to explore the underlying mechanisms of protein dynamics. Previous studies have revealed changes in protein and mRNA expression levels,^18^^,^^19^^,^^20^ gain and/or loss of PPIs^21^ and protein-DNA interactions.^22^ In this study, we reveal that protein redistribution is an additional source of paralog retention, whereby proteins respond to the loss of their paralog by changing their subcellular localization or protein abundance.

Recent advances in phenomic screening and analysis approaches^59^ are opening new avenues for increasing the scale of these screens and the resolution to the single-cell level. Advancements in genetic manipulation in human cells are also paving the way for extending these findings to other systems. Our study demonstrates the potential of such technologies in addressing fundamental questions in the broad field of genome evolution.

### Limitations of the study

The limitation of the current study is the relatively small sample size of our examined paralog set. In the future, small-scale duplicates should be added to increase the sample size allowing us to subdivide the data further and add finer comparative analyses related to redistributed paralogs. Further refinement of the redistribution scoring strategy using deep neural network features, which was used as a complementary metric to visual inspection of subcellular localization changes and quantified protein abundance changes, would increase its sensitivity by capturing modest protein abundance changes. In addition, the extent of responsiveness is likely underestimated since paralog responsiveness is sometimes revealed in specific growth conditions, which require that gene function. Thus, expanding the repertoire of conditions would provide a more complete understanding of the extent of paralog responsiveness.

## Resource availability

### Lead contact

Further information and requests for resources and reagents should be directed to and will be fulfilled by the lead contact, Elena Kuzmin (elena.kuzmin@concordia.ca).

### Materials availability

Yeast strains generated in this study are available upon request.

### Data and code availability

• Tables S1, S2, S3, S4 and S5; Data S1 and S2 were also deposited in Mendeley Data, V1, https://doi.org/10.17632/zx9xw7w28f.1.
• The primary data are available through a searchable website PARPAL (https://parpal.c3g-app.sd4h.ca/) and the raw microscopy images are available upon request.
• Source code for the analysis and the visualizations is available on GitHub at https://github.com/Kuzmin-Lab-code/yeast-paralog-redistribution.

## Acknowledgments

We thank Malcolm Whiteway and Kuzmin laboratory members for discussions and critical comments on the manuscript. We thank Chris Law and the Center for Microscopy and Cellular Imaging at Concordia University for guidance on microscopy. Supported by Canada Research Chair program grant (CRC2021-00031 to E.K.), Canada Foundation for Innovation grant (CFI 41563 to E.K.), Natural Sciences and Engineering Research Council grant (RGPIN-2022-04674 to E.K.), Wellcome Trust (220540/Z/20/A to L.P.) and Revvity Inc. (VLTAT19682 to D.F. and L.P.), the National Institutes of Health (R01HG005853 to B.J.A. and C.B.), and the Canadian Institutes of Health Research (PJT-180259 to B.J.A.). Computing resources and data storage services were partially provided by the High Performance Computing Center of the University of Tartu and the Digital Research Alliance of Canada.

## Author contributions

Conceptualization: R.D. and E.K.; methodology and investigation: R.D., M.P., B.M.G., V.P., D.F., H.F., E.S., and E.K.; formal analysis: R.D., M.P., B.M.G., V.P., D.F., C.B., B.J.A., L.P. and E.K.; resources: K.W., O.K., and B.G.; database development: G.Z., F.L., V.P., and G.B.; writing – original draft: R.D. and E.K.; writing – review and editing: R.D., M.P., B.M.G., V.P., D.F., E.S., H.F., C.B., B.J.A., L.P., and E.K.; supervision: E.K.; funding acquisition: C.B., B.J.A., L.P., and E.K.

## Declaration of interests

The authors declare no competing interests.

## STAR★Methods

### Key resources table

**Table:** 

REAGENT or RESOURCE	SOURCE	IDENTIFIER
**Antibodies**		

GFP anti-mouse	Takara	Cat#632381; RRID:AB_2313808
ZWF1 anti-rabbit	Sigma	Cat#A9521; RRID:AB_258454
IgG, HRP-linked anti-mouse	NEB	Cat#7076S
IgG, HRP-linked anti-rabbit	NEB	Cat#7074S

**Oligonucleotides**		

All oligonucleotides are listed in the STAR Methods section	This paper	N/A

**Biological Samples**		

Single gene deletion strains (SN query strain collection)	Costanzo et al. 2016	https://doi.org/10.1126/science.aaf1420
Strains from the GFP collection	Huh et al. 2003	https://doi.org/10.1038/nature02026
LocRed strains	This paper	N/A

**Deposited Data**		

Tables S1, S2, , S3, S4 and S5 and Data S1 and S2	Mendeley	Mendeley Data, V1, https://doi.org/10.17632/zx9xw7w28f.1

**Software and Algorithms**		

PyTorch (1.4.0)	https://pypi.org/project/torch/	https://doi.org/10.48550/arXiv.1912.01703
scikit-learn (0.5.3)	https://pypi.org/project/scikit-learn/	https://doi.org/10.48550/arXiv.1201.0490
htsimaging (1.0.5)	https://pypi.org/project/htsimaging/	https://zenodo.org/doi/10.5281/zenodo.3697134
scikit-image (0.16.2)	https://pypi.org/project/scikit-image/	van der Walt et al. 2014
ND2	https://pypi.org/project/nd2	N/A
roux (0.1.1)	https://pypi.org/project/roux/	https://zenodo.org/doi/10.5281/zenodo.2682670
statsmodels (0.13.2)	https://pypi.org/project/statsmodels/	https://doi.org/10.25080/Majora-92bf1922-011
scipy (1.7.3)	https://pypi.org/project/scipy/	https://doi.org/10.1038/s41592-019-0686-2
numpy (1.21.6)	https://pypi.org/project/numpy/	https://doi.org/10.1038/s41586-020-2649-2
networkx (2.6.3)	https://pypi.org/project/networkx/	http://conference.scipy.org.s3-website-us-east-1.amazonaws.com/proceedings/scipy2008/paper_2/
pandas (1.3.5)	https://pypi.org/project/pandas/	https://doi.org/10.5281/zenodo.8239932
matplotlib (3.5.1)	https://pypi.org/project/matplotlib/	https://doi.org/10.1109/MCSE.2007.55
seaborn (0.12.2)	https://pypi.org/project/seaborn/	https://doi.org/10.21105/joss.03021
DeepLoc - 2.0	https://services.healthtech.dtu.dk/services/DeepLoc-2.0/	https://doi.org/10.1093/nar/gkac278
EMBOSS (6.5.7.0)	https://bioconda.github.io/recipes/emboss/README.html	N/A
Fiji (v.2.3)	NIH	https://imagej.net/software/fiji/downloads

### Experimental model and study participant details

#### Strain maintenance

All query strains were maintained on YEPD media (1% yeast extract, 2% peptone, 2% glucose, 0.012% adenine) supplemented with 100 μg/mL nourseothricin (clonNAT) (Werner Bioagents) and array strains on YEPD supplemented with 200 μg/mL geneticin (G418) (Agri-Bio). Single gene deletion strains were obtained from the SN query strain collection^43^ and GFP strains were obtained from the GFP collection.^34^

#### Query strain construction

We constructed and successfully screened 328 yeast query strains, each harboring a GFP fusion in wild-type or deletion background of its respective WGD paralog (Table S1). All duplicated genes originating from whole genome duplication^33^ that were in the O’Shea collection,^34^ https://yeastgfp.yeastgenome.org were examined. Paralog pairs were selected that exhibited different subcellular localizations or that were given an “ambiguous” annotation. Those that were "ambiguous" were examined in the CYCLoPs data^29^ and those with distinct subcellular localization patterns were selected.

Strains from the GFP collection^34^ with the following genotype were used: *MAT***a** paralog:GFP::HIS3MX6 *his3*Δ*1 leu2*Δ*0 met15*Δ*0 ura3*Δ*0.* To construct kanR GFP strains, they were marker switched from *HIS3* to *kanMX4* by PCR-mediated recombination strategy using high-efficiency LiAc transformation. p116 plasmid was used to PCR amplify *kanMX4.* This plasmid is ampicillin resistant and was cultured in 2YT + 120 μg/mL ampicillin. Primers that were used for *kanMX4* amplification were:

MX4-F (5′ 3′): *ACATGGAGGCCCAGAATACCCT*.

MX4-R (5′ 3′): *CAGTATAGCGACCAGCATTCAC*.

Marker switch was confirmed by replica plating on SD-his to confirm His^−^ phenotype of the kanR transformants. The resulting kanR GFP strains were introduced into the desired deletion background SN# (*MAT****α*** *paralog*Δ*::natMX4 can1*Δ*::STE2pr-Sp_his5 lyp1*Δ *his3*Δ*1 leu2*Δ*0 ura3*Δ*0 met15*Δ*0 LYS2+*) or the control strain Y8835 (*MAT****α*** *ura3*Δ*::natMX4 can1*Δ*::STE2pr-Sp_his5 lyp1*Δ *his3*Δ*1 leu2*Δ*0 ura3*Δ*0 met15*Δ*0 LYS2+*) by SGA.^60^^,^^61^^,^^62^ Three control pairs were included in which random paralogs were paired together. Briefly, the strains were crossed, sporulated and *MAT***a** meiotic progeny were selectively germinated using *STE2pr-Sp_his5* marker.

Final strains were of the following genotypes (Table S1).

(1) *PAR1::GFP::kanMX4 ura3Δ::natMX4 can1Δ::STE2pr-Sp_his5 lyp1Δ his3Δ1 leu2Δ0 ura3Δ0 met15Δ0 LYS2+*
(2) *PAR1::GFP::kanMX4 par2Δ:natMX4 can1Δ::STE2pr-Sp_his5 lyp1Δ his3Δ1 leu2Δ0 ura3Δ0 met15Δ0 LYS2+*
(3) *PAR2::GFP::kanMX4 ura3Δ::natMX4 can1Δ::STE2pr-Sp_his5 lyp1Δ his3Δ1 leu2Δ0 ura3Δ0 met15Δ0 LYS2+*
(4) *PAR2::GFP::kanMX4 par1Δ::natMX4 can1Δ::STE2pr-Sp_his5 lyp1Δ his3Δ1 leu2Δ0 ura3Δ0 met15Δ0 LYS2+*

#### Microscopy

Cells were prepared for imaging and imaged as described in detail previously.^60^^,^^63^ Briefly, cells were grown to saturation in 200 mL SDmsg medium (0.1% monosodium glutamate, 0.17% yeast nitrogen base without amino acids and without ammonium sulfate, 2% glucose, 0.15 g/L methionine) containing 200 μg/mL G418 and 100 μg/mL clonNAT in 96-well beaded microplates, then diluted in 800 mL SDmsg low fluorescence medium (with 0.17% yeast nitrogen base without amino acids and without ammonium sulfate and without riboflavin and folic acid) containing 200 μg/mL G418 and 100 μg/mL clonNAT in beaded deep-well blocks and grown overnight to early log phase. All strains were grown at 30°C. Cells at ∼0.2–0.4 OD600/mL were transferred to a 384-well PerkinElmer Ultra imaging plate and left to settle for 10 min before imaging. Four images per well, each containing fifty to a hundred cells, were taken in a single plane using an automated spinning disk confocal microscope (Evotec Opera, PerkinElmer) with a 60× water-immersion objective.

#### Image analysis

##### Segmentation

###### Annotation processing

We processed ground truth object annotation to provide a segmentation neural network with the right objective to efficiently separate individual cells in clumps. To transform object segmentation into binary data and distinguish between the individual stencils, we eroded each stencil with the minimal cross, i.e., we claim the pixel to be background if any of its four neighbors is from the background. After erosion, we performed a Euclidean distance transformation of the binary segmentation by labeling each pixel with the distance to the nearest background pixel using SciPy.^64^ The transformed result was clipped at 20 and scaled between 0 and 1. Finally, we took a weighted average between binary segmentation and the distance map with a coefficient of 0.8, i.e., border pixels of distance-transformed stencils obtained a value of 0.8, which gradually increased to 1 maximum toward the object center (Figure S8). Such target transformation forced the segmentation algorithm to focus primarily on object centers and effectively separate the cells.

###### Image processing

Before further transformations, we calculated a reference background image and subtracted it from all the images in the respective dataset. To do so, noise in each coordinate was calculated as a median pixel value by this coordinate across all the images in the dataset. This was done to mitigate an uneven illumination pattern as well as the readout noise across the microscope’s field of view.

Protein abundance varies greatly depending on the protein localization and query gene. Thus, the detected signal range in images obstructs segmentation networks from generalization and efficient convergence. To mitigate this problem, we applied a logarithmic transformation to the base of *e* to all the images and standardized the pixel values in each image to mean 0 and standard deviation 1.

$$x^{\prime }=\log\left(x+1\right)$$

$$x^{\prime \prime }=\frac{x^{\prime }-\mu }{\sigma }$$

###### Neural network architecture and training for segmentation

For segmentation, we trained a fully convolutional U-Net neural network architecture^65^ from the Segmentation models PyTorch library.^66^ The training set was obtained from previously manually annotated images of yeast cells that were imaged on the same microscope.^67^ Each annotation contained a pixel-level instance segmentation of yeast cells in the image. We utilized ResNet-34^68^ backbone as a network encoder. It consists of a single convolutional layer with 64 filters 7 × 7, followed by 16 residual units in blocks of 3, 4, 6, and 3 with 64, 128, 256, and 512 filters 3 × 3 respectively. Each residual unit consists of a layer sequence BN-ReLU-Conv-BN-ReLU-Conv with additively connected input and output. The first convolutional layer and each group of residual blocks are followed by a max-pooling layer and are considered to be a U-Net level. The encoder is followed by the decoder. The decoder consists of five levels Conv-ReLU-Conv-ReLU with 64 filters 3 × 3 per convolutional layer. Each encoder level is connected to the respective decoder level by a skip-connection. The final decoder layer outputs a single-channel probability map through the sigmoid activation function.

We implemented the network with the PyTorch deep learning library^69^ in Python. Network parameters were initialized as proposed previously.^70^ We used the transformed distance maps (see Annotation processing) as training targets and optimized binary cross-entropy loss for the network’s predictions. The network was trained in the PyTorch Lightning framework^71^ for 200 epochs with a batch size of 32. Adam optimization^72^ with default hyperparameters was used. We utilized the cosine annealing learning rate schedule^69^^,^^73^ from an initial value 3 × 10^−4^ to 1 × 10^−7^.

For each image in a training batch, we randomly cropped patches 256 × 256 pixels from the original images and augmented them with random flipping and rotation by (0, 90, 180, 270) degrees. Additionally, each image was scaled and shifted by factors, sampled uniformly from ranges [0.5, 2) and [-1, 1) respectively. For the inference, we padded single full-size images with reflection until divisibility by 32 to adhere to network architecture requirements; outputs in padded areas were not analyzed. We trained the network using 2246 images and validated its performance with 250 images after each epoch, using 1 validation image per gene label. We saved the best weights by a validation cross-entropy loss and used them in our following experiments.

###### Post-processing algorithm

Individual cells are not distinguished perfectly in the segmentation network’s output. To split the clumps, we used the Watershed algorithm from the scikit-image library.^74^ The network predictions thresholded at 0.8 were used as watershed seeds, and a threshold of 0.5 was used to obtain object boundaries. After the watershed, we removed any objects smaller than 256 and larger than 8192 pixels by area.

#### Neural network architecture and training for feature extraction

We considered each object in the output segmentation map to be a cell. From each full-size image, we extracted cell frames of size 64 × 64 pixels around all detected cell centers. We then trained a neural network classifier to distinguish cell frames between all 182 GFP-tagged proteins in the wild-type background. Many of the genes share a subcellular localization, so we did not utilize the last dense classification layer of the network after the training. Instead, for each cell frame, we extracted a 128-dimensional feature vector from the penultimate pooling layer. These features do not directly map to the perceived cell properties, such as size, etc. However, they should provide sufficiently compressed information about the protein subcellular localization.

For feature extraction, we trained a ResNet-18 neural network architecture. It consists of a single convolutional layer with 16 filters 3 × 3, followed by 4 residual blocks of 2 residual units each with 16, 32, 64, and 128 filters 3 × 3, respectively. Each residual unit consists of a layer sequence BN-ReLU-Conv-BN-ReLU-Conv with additively connected input and output. Feature extractor is concluded by a global average pooling layer, which yields 128 features for each single cell. During the training, these features were passed to the dense classification layer with 182 outputs followed by a softmax activation function. We used simple image augmentations from the Albumentations library^75^: random horizontal and vertical flipping, shifting, scaling, and rotation with default parameters.

We implemented the network with the PyTorch deep learning library^69^ in Python. Network parameters were initialized as proposed previously.^70^ We used encoded gene labels as training targets and optimized categorical cross-entropy loss for the network’s predictions. The network was trained in the PyTorch Lightning framework^71^ for 200 epochs with a batch size of 512. Adam optimization^72^ with default hyperparameters was used. We utilized a previously defined cosine annealing learning rate schedule. We used all the wild-type cell frames from Field 4 for validation (∼25% of the total number) and the rest of the frames from Fields 1–3 for training. We did not distinguish between replicates at this stage.

#### Filtering of the imaging data

The proteins whose localization was mismatched with the known localizations,^29^ images containing abnormalities, artifacts, and excessively high heterogeneity; as well as the replicates containing very low cell numbers were removed from the dataset. In total, of 182 GFP-tagged proteins, 9 paralog pairs (18 GFP-tagged proteins) were removed from the dataset based on this filtering procedure, resulting in 82 paralog pairs (164 GFP-tagged proteins) in the final dataset.

#### Redistribution analysis

We derived a single metric, termed the redistribution score, which captures changes across all cells for all 128 features which are obtained from the deep neural network to gauge the extent of change in protein distribution between the wild type and deletion backgrounds of their paralogs. For this purpose, we calculated an arithmetic mean across all the cells in three replicates for each feature. Then, we obtain a single 128-dimensional centroid point for each GFP-tagged protein in a given background. The redistribution score is then the Euclidean distance (length of the vector) between the centroid for a protein in a wild-type background of its paralog and the centroid of this protein in the deletion background of its paralog.

To visually represent the redistribution in the 128-dimensional feature space, we utilized the Principal Component Analysis (PCA) method for dimensionality reduction. We first *Z* score normalized the feature values in order to ensure that the variance within them does not confound the PCA. Then we performed PCA to obtain the dimensional principal components (PCs). In the visualizations, we show the first two PCs because they collectively account for the largest portion of the variation in the data. The relative positioning of the PCA cluster corresponding to the deletion background with respect to the WT background—shown by arrows—provides the representation of the paralog redistribution. We note that the PCA serves only the purpose of dimensionality reduction for visual representation. The redistribution scores are calculated from the features values as described above.

We manually inspected every image to identify whether the deletion of the paralog resulted in an change to protein abundance evident by a decrease or an increase to the brightness of the green fluorescence in the microscopy image or a change in subcellular localization evident by a difference to the pattern of the green fluorescence signal in the cells in the microscopy image. Visual inspection provided us an additional level of confidence in the redistribution scores that we obtained from the machine learning approach. The threshold for classifying the paralogs based on the redistribution score was calculated using AUC-ROC analysis (Figure S3A). In this analysis, 12 visually inspected paralogs that showed relocalization and/or relative abundance change were used as true cases, and negative controls i.e., the six paralogs that were paired randomly were used as the false cases. The threshold providing the minimal False Positive Rate (FPR) was considered.

#### Protein abundance quantification

We quantified the protein abundance by measuring raw pixel intensities in cell frames. For both conditions, we measured mean raw pixel intensity across all the pixels in all the frames in the condition, which we refer to as the abundance score. For calculating the relative abundance changes, we first log-transformed (base = 2) the abundance scores with a pseudocount of 1 and then calculated the difference between the WT and deletion backgrounds. The statistical significance of the differences were calculated using two-sided Mann-Whitney U test, and corrected for multiple testing using Benjamini/Hochberg method.

#### Experimental validation of relocalization

Strains of two redistributed paralog pairs (*GGA1-GGA2* and *HMS2-SKN7*) representing cases of compensation and dependency were re-imaged to ensure reproducibility using Leica Confocal Microscope, 40X.

#### Paralog features

##### Protein-protein interactions

PPI data were downloaded from BioGRID^76^ (downloaded on 2022-06-27). The data was filtered to keep only physical ‘Experimental System Type’s and interactions from high-throughput studies.^77^^,^^78^^,^^79^^,^^80^^,^^81^

##### Genetic interactions

Digenic interaction scores were obtained from a previous study.^43^ The subsets of the interactions i.e., lenient (*p* < 0.05), intermediate (*p* < 0.05 and interaction score |ε| > 0.08), and stringent (*p* < 0.05 and ε > 0.16 or ε < −0.12) were obtained by applying the previously defined thresholds. Trigenic interaction fraction classes were obtained from a previous study.^17^

##### Network features

The degree and the shortest path lengths were calculated using the networkx Python package.^82^

##### Colocalization

The protein localizations primarily obtained from^29^ were complemented with ones from another study.^83^ The colocalization was classified based on the Jaccard index

$$\left(\frac{intersection}{union}\times 100\right)\geq 50$$

##### Morphological features

Cell features related to cellular morphology were quantified. Area denotes cell area; convex area denotes the area of the convex hull image; equivalent diameter denotes diameter of a circle with the same area as the cell; perimeter denotes cell perimeter; eccentricity denotes elongation of the equivalent ellipse; major/minor axis length denotes major/minor axis length of equivalent ellipse; solidity denotes ratio of pixels in the cell to pixels in convex area; orientation denotes cell orientation; Euler number denotes number of connected regions subtracted by number of holes. Spearman correlation coefficient was measured between these features and those derived based on the neural network. Statistical significance was assessed using a two-sided Mann-Whitney U-test.

#### Prediction of the relocalization of alternate isoforms

The sequence of the alternate protein isoform of Cue4 protein was obtained from a previous study.^46^ The Translational Initiation Site (TIS) profiling data for the vegetative exponential phase (SRR11777267) was used to visualize the read depth along the length of Cue4 in Figure 7D. Using the protein sequences of Cue4 and its alternate protein isoform, their localizations were predicted using DeepLoc2.0.^84^

#### Validation of Cue4-GFP redistribution

##### Microscopy

The strain Y109 *(MAT***a** *CUE4::GFP::kanMX4 cue1Δ::natMX4)* was reconstructed by PCR mediated-deletion, which replaced *CUE1* with a *natMX4* cassette using *MAT***a** *CUE4::GFP::kanMX4* starting strain. *CUE1* gene deletion was confirmed using colony PCR. Both Y109 and LocRed-6 (*MAT*a C*UE4::GFP::kanMX4 ura3Δ::natMX4)* strains were crossed to the strain Y110 harboring *SPF1-mScarlet,* an ER reporter (*MAT* α *SPF1-mScarlet-HYG*). Diploid strains were sporulated and tetrad dissected to select for the genotype of interest (NAT^R^, G418^R^, HYG^R^). The resulting strains (Y122 and Y123) were grown in YEPD to mid-log phase at 30^o^C. Live cells were rinsed once with water and once with low-fluorescent media.^85^ Then the strains were imaged in low-fluorescent media using the Nikon Eclipse TiE inverted C2 confocal microscope at 100× oil immersion objective with filters for GFP (488/10x + 525/50m) and mScarlet (561/10x + 630/75m). DIC, GFP, and mScarlet were imaged sequentially. Refer to Table S1 for complete strain genotype details.

The individual channels from the raw images (Nikon NIS-Elements ND2 format) were separated using nd2 Python package (https://pypi.org/project/nd2). The images from the channel with the GFP intensity were used to carry out the cell segmentation, using YeastSpotter.^86^ The single cells were labeled using scikit-image^74^ to assign a unique identifier to each cell. On average 50 cells per strain were quantified in three independent replicates. The cells at the edges of the images, within the median major axis length of the cells, were removed. The raw protein abundance per cell was calculated as the median value of the intensity, which was then divided by the intensity of the background to calculate the normalized abundance per cell, using htsimaging Python package.^87^ Within each replicate, the images with extreme background intensity were discarded using the mean+/−stdev as thresholds. The images from the red channel containing the intensity for the ER reporter, Spf1-mScarlet, were used to identify the locations of the ER within the cells. Within each cell, the pixels with intensity ≥0.975 quantile were marked as the locations of the ER, whereas the ones with intensity <0.973 quantile were marked as cytoplasmic. The cells with less than 100 ER pixels were discarded. Similar to the calculation of the cell-wise protein abundance, the protein abundances at the ER and cytoplasmic locations were calculated as the median value of the intensity at these locations divided by the background intensity of the image.^87^

Imaging data were analyzed using open-source Python packages such as scikit-image^74^ and htsimaging.^87^ The statistical analysis was carried out using scipy,^64^ statsmodels,^88^ scikit-learn,^89^ numpy^90^ and pandas.^91^ The network analysis was carried out using networkx.^82^ The data visualization was done using matplotlib^92^ and seaborn.^93^ The Python package roux^94^ was used for both data analysis and visualization. The versions of these and other tools used in the analysis are provided along with the source code.

##### Protein extraction and Western Blot

Cue4-GFP wildtype (LocRed-6) and Cue4-GFP *cue1*Δ (Y109) cells were grown overnight in Synthetic Complete media supplemented with 100 μg/mL nourseothricin (cloNAT) (Werner Bioagents) and 200 μg/mL geneticin (G418) (ThermoFisher, 11811098). Next, a subculture with a measured OD600 of between 0.6 and 0.8 was harvested at 4°C. The cells were washed in 1 mL of water then resuspended in 1 mL 0.288 M NaOH and incubated on ice for 10 min. To the 0.288 M NaOH, 75 μL of 100% Trichloroacetic acid (MilliporeSigma, T0699) was added prior to vortexing and incubated on ice for 10 min. The NaOH-Trichloroacetic acid supernatant was removed and 100 μL of HU buffer with DTT (8 M Urea, 5% SDS, 200 mM Tris-Cl pH 6.8, 0.1 mM EDTA, 15 mg/mL DTT, and bromophenol blue) was added to the precipitated proteins and heated for 15 min at 65°C. The samples were centrifuged and 12 μL of cell lysates were used for Western Blot analysis with a 10% precast polyacrylamide gel (Bio-Rad, 4561033EDU) and running buffer (2.5 mM Tris, 19.2 mM Glycine, and 0.1% SDS). The proteins were transferred to PVDF membrane with Trans-Blot Turbo Buffer (Bio-Rad, 1704274) and the accompanying Bio-Rad transfer system. Protein detection was performed using ECL chemiluminescent substrate (Bio-Rad, 1705062) and the Amersham Imager 600 (GE Healthcare) following the incubation with mouse anti-GFP primary antibody (Takara, 632381) and anti-mouse HRP-linked secondary antibody (NEB, 7076S) as well as rabbit anti-ZWF1 primary antibody (Sigma, A9521) and anti-rabbit HRP-linked secondary antibody (NEB, 7074S). The quantification of protein abundance was performed using Fiji software.

### Quantification and statistical analysis

Statistical parameters, including number of biological replicates, sample sizes, algorithm parameters are reported in the method details and the figure legends.

## Supplemental information

Document S1. Figures S1–S8

Table S1. Yeast strains and plasmids used in this study (A) Yeast query strain list including strain IDs, paralog ORFs, and gene names. (B) Genotypes for yeast query strains. (C) Plasmid IDs and descriptions.

Table S2. Protein abundance (A) Description of each column. (B) Yeast query strain list including strain IDs, paralog ORFs, gene names, protein abundance mean, and protein abundance of each replicate.

Table S3. Manual inspection of paralog pairs and negative controls (A) Description of each column. (B) Results of manual inspection with redistribution score provided for comparison.

Table S4. The redistribution, relative abundance change and relocalization (A) Description of each column. (B) Paralog pair ORFs and gene names; GFP tagged protein ORF and gene name; redistribution score and whether it met the 4.73 threshold (true if redistribution score ≥4.73 and false if redistribution score <4.73); relative abundance change log_2_ fold change; relative abundance change log_2_ fold change q-value; relative abundance change type ns if not significant, compensation if log_2_ fold change ≥0.2, *q* < 0.05, dependency if log_2_ fold change ≤ −0.2, *q* < 0.05; relocalization type as assessed by visual inspection with the corresponding description.

Table S5. Features of paralogs (A) Description of each column. (B) Paralog pair ORFs and gene names; protein-protein interaction (PPI) interactors shared; genetic interaction (GI) shortest path length; PPI shortest path length; Negative GI; Trigenic interaction fraction class; GI interactors colocalize with sister paralog gene1; GI interactors colocalize with sister paralog gene2; PPI interactors colocalize with sister paralog gene1; PPI interactors colocalize with sister paralog gene2.

Data S1. Deep neural network features of single cells Gene class paralogs or random pairs; strain ID; gene name; genetic background of wild-type or deletion of its paralog; replicate number; cell ID; feature 1–128.

Data S2. Protein abundance per single cell Gene class paralogs or random pairs; strain ID; gene name; genetic background of wild-type or deletion of its paralog; replicate number; cell ID; protein abundance.

## References

[1] Kuzmin E., Taylor J.S., Boone C. (2022). Retention of duplicated genes in evolution. Trends Genet..

[2] Spring J. (1997). Vertebrate evolution by interspecific hybridisation--are we polyploid?. FEBS Lett..

[3] Fisher K.J., Buskirk S.W., Vignogna R.C., Marad D.A., Lang G.I. (2018). Adaptive genome duplication affects patterns of molecular evolution in Saccharomyces cerevisiae. PLoS Genet..

[4] Hilario E., Gogarten J.P. (1993). Horizontal transfer of ATPase genes--the tree of life becomes a net of life. Biosystems.

[5] Hughes A.L., Friedman R., Ekollu V., Rose J.R. (2003). Non-random association of transposable elements with duplicated genomic blocks in Arabidopsis thaliana. Mol. Phylogenet. Evol..

[6] Force A., Lynch M., Pickett F.B., Amores A., Yan Y.L., Postlethwait J. (1999). Preservation of duplicate genes by complementary, degenerative mutations. Genetics.

[7] Kondrashov F.A., Kondrashov A.S. (2006). Role of selection in fixation of gene duplications. J. Theor. Biol..

[8] Nowak M.A., Boerlijst M.C., Cooke J., Smith J.M. (1997). Evolution of genetic redundancy. Nature.

[9] Ohno S. (1970). Evolution by Gene Duplication.

[10] Gu Z., Steinmetz L.M., Gu X., Scharfe C., Davis R.W., Li W.H. (2003). Role of duplicate genes in genetic robustness against null mutations. Nature.

[11] Conant G.C., Wagner A. (2004). Duplicate genes and robustness to transient gene knock-downs in Caenorhabditis elegans. Proc. Biol. Sci..

[12] Hanada K., Kuromori T., Myouga F., Toyoda T., Li W.H., Shinozaki K. (2009). Evolutionary persistence of functional compensation by duplicate genes in Arabidopsis. Genome Biol. Evol..

[13] De Kegel B., Ryan C.J. (2019). Paralog buffering contributes to the variable essentiality of genes in cancer cell lines. PLoS Genet..

[14] Dandage R., Landry C.R. (2019). Paralog dependency indirectly affects the robustness of human cells. Mol. Syst. Biol..

[15] Costanzo M., Kuzmin E., van Leeuwen J., Mair B., Moffat J., Boone C., Andrews B. (2019). Global Genetic Networks and the Genotype-to-Phenotype Relationship. Cell.

[16] VanderSluis B., Bellay J., Musso G., Costanzo M., Papp B., Vizeacoumar F.J., Baryshnikova A., Andrews B., Boone C., Myers C.L. (2010). Genetic interactions reveal the evolutionary trajectories of duplicate genes. Mol. Syst. Biol..

[17] Kuzmin E., VanderSluis B., Nguyen Ba A.N., Wang W., Koch E.N., Usaj M., Khmelinskii A., Usaj M.M., van Leeuwen J., Kraus O. (2020). Exploring whole-genome duplicate gene retention with complex genetic interaction analysis. Science.

[18] Kafri R., Levy M., Pilpel Y. (2006). The regulatory utilization of genetic redundancy through responsive backup circuits. Proc. Natl. Acad. Sci. USA.

[19] DeLuna A., Springer M., Kirschner M.W., Kishony R. (2010). Need-Based Up-Regulation of Protein Levels in Response to Deletion of Their Duplicate Genes. PLoS Biol..

[20] Burga A., Casanueva M.O., Lehner B. (2011). Predicting mutation outcome from early stochastic variation in genetic interaction partners. Nature.

[21] Diss G., Gagnon-Arsenault I., Dion-Coté A.M., Vignaud H., Ascencio D.I., Berger C.M., Landry C.R. (2017). Gene duplication can impart fragility, not robustness, in the yeast protein interaction network. Science.

[22] Gera T., Jonas F., More R., Barkai N. (2022). Evolution of binding preferences among whole-genome duplicated transcription factors. eLife.

[23] Klaasen S.J., Truong M.A., van Jaarsveld R.H., Koprivec I., Štimac V., de Vries S.G., Risteski P., Kodba S., Vukušić K., de Luca K.L. (2022). Nuclear chromosome locations dictate segregation error frequencies. Nature.

[24] Manolson M.F., Wu B., Proteau D., Taillon B.E., Roberts B.T., Hoyt M.A., Jones E.W. (1994). STV1 gene encodes functional homologue of 95-kDa yeast vacuolar H(+)-ATPase subunit Vph1p. J. Biol. Chem..

[25] Preston R.A., Murphy R.F., Jones E.W. (1989). Assay of vacuolar pH in yeast and identification of acidification-defective mutants. Proc. Natl. Acad. Sci. USA.

[26] Manolson M.F., Proteau D., Preston R.A., Stenbit A., Roberts B.T., Hoyt M.A., Preuss D., Mulholland J., Botstein D., Jones E.W. (1992). The VPH1 gene encodes a 95-kDa integral membrane polypeptide required for in vivo assembly and activity of the yeast vacuolar H(+)-ATPase. J. Biol. Chem..

[27] Tkach J.M., Yimit A., Lee A.Y., Riffle M., Costanzo M., Jaschob D., Hendry J.A., Ou J., Moffat J., Boone C. (2012). Dissecting DNA damage response pathways by analysing protein localization and abundance changes during DNA replication stress. Nat. Cell Biol..

[28] Breker M., Gymrek M., Schuldiner M. (2013). A novel single-cell screening platform reveals proteome plasticity during yeast stress responses. J. Cell Biol..

[29] Chong Y.T., Koh J.L.Y., Friesen H., Duffy S.K., Cox M.J., Moses A., Moffat J., Boone C., Andrews B.J. (2015). Yeast Proteome Dynamics from Single Cell Imaging and Automated Analysis. Cell.

[30] Kraus O.Z., Grys B.T., Ba J., Chong Y., Frey B.J., Boone C., Andrews B.J. (2017). Automated analysis of high-content microscopy data with deep learning. Mol. Syst. Biol..

[31] Lu A.X., Chong Y.T., Hsu I.S., Strome B., Handfield L.F., Kraus O., Andrews B.J., Moses A.M. (2018). Integrating images from multiple microscopy screens reveals diverse patterns of change in the subcellular localization of proteins. eLife.

[32] Koh J.L.Y., Chong Y.T., Friesen H., Moses A., Boone C., Andrews B.J., Moffat J. (2015). CYCLoPs: A Comprehensive Database Constructed from Automated Analysis of Protein Abundance and Subcellular Localization Patterns in Saccharomyces cerevisiae. G3 (Bethesda).

[33] Byrne K.P., Wolfe K.H. (2005). The Yeast Gene Order Browser: combining curated homology and syntenic context reveals gene fate in polyploid species. Genome Res..

[34] Huh W.K., Falvo J.V., Gerke L.C., Carroll A.S., Howson R.W., Weissman J.S., O'Shea E.K. (2003). Global analysis of protein localization in budding yeast. Nature.

[35] Ho B., Baryshnikova A., Brown G.W. (2018). Unification of Protein Abundance Datasets Yields a Quantitative Saccharomyces cerevisiae Proteome. Cell Syst..

[36] Kim S.H., Yi S.V. (2006). Correlated asymmetry of sequence and functional divergence between duplicate proteins of Saccharomyces cerevisiae. Mol. Biol. Evol..

[37] Boles E., Liebetrau W., Hofmann M., Zimmermann F.K. (1994). A family of hexosephosphate mutases in Saccharomyces cerevisiae. Eur. J. Biochem..

[38] Jones A.L., Quimby B.B., Hood J.K., Ferrigno P., Keshava P.H., Silver P.A., Corbett A.H. (2000). SAC3 may link nuclear protein export to cell cycle progression. Proc. Natl. Acad. Sci. USA.

[39] Farkas Z., Kovács K., Sarkadi Z., Kalapis D., Fekete G., Birtyik F., Ayaydin F., Molnár C., Horváth P., Pál C., Papp B. (2022). Gene loss and compensatory evolution promotes the emergence of morphological novelties in budding yeast. Nat. Ecol. Evol..

[40] Zhdankina O., Strand N.L., Redmond J.M., Boman A.L. (2001). Yeast GGA proteins interact with GTP-bound Arf and facilitate transport through the Golgi. Yeast.

[41] Raitt D.C., Johnson A.L., Erkine A.M., Makino K., Morgan B., Gross D.S., Johnston L.H. (2000). The Skn7 response regulator of Saccharomyces cerevisiae interacts with Hsf1 in vivo and is required for the induction of heat shock genes by oxidative stress. Mol. Biol. Cell.

[42] Janiak-Spens F., Cook P.F., West A.H. (2005). Kinetic analysis of YPD1-dependent phosphotransfer reactions in the yeast osmoregulatory phosphorelay system. Biochemistry.

[43] Costanzo M., VanderSluis B., Koch E.N., Baryshnikova A., Pons C., Tan G., Wang W., Usaj M., Hanchard J., Lee S.D. (2016). A global genetic interaction network maps a wiring diagram of cellular function. Science.

[44] Shih S.C., Prag G., Francis S.A., Sutanto M.A., Hurley J.H., Hicke L. (2003). A ubiquitin-binding motif required for intramolecular monoubiquitylation, the CUE domain. EMBO J..

[45] Bagola K., von Delbrück M., Dittmar G., Scheffner M., Ziv I., Glickman M.H., Ciechanover A., Sommer T. (2013). Ubiquitin binding by a CUE domain regulates ubiquitin chain formation by ERAD E3 ligases. Mol. Cell.

[46] Eisenberg A.R., Higdon A.L., Hollerer I., Fields A.P., Jungreis I., Diamond P.D., Kellis M., Jovanovic M., Brar G.A. (2020). Translation Initiation Site Profiling Reveals Widespread Synthesis of Non-AUG-Initiated Protein Isoforms in Yeast. Cell Syst..

[47] Li J., Yuan Z., Zhang Z. (2010). The Cellular Robustness by Genetic Redundancy in Budding Yeast. PLoS Genet..

[48] Genuth N.R., Barna M. (2018). The Discovery of Ribosome Heterogeneity and Its Implications for Gene Regulation and Organismal Life. Mol. Cell.

[49] Komili S., Farny N.G., Roth F.P., Silver P.A. (2007). Functional specificity among ribosomal proteins regulates gene expression. Cell.

[50] Segev N., Gerst J.E. (2018). Specialized ribosomes and specific ribosomal protein paralogs control translation of mitochondrial proteins. J. Cell Biol..

[51] Marino-Ramirez L., Hu J.C. (2002). Isolation and mapping of self-assembling protein domains encoded by the Saccharomyces cerevisiae genome using lambda repressor fusions. Yeast.

[52] Kaluarachchi Duffy S., Friesen H., Baryshnikova A., Lambert J.P., Chong Y.T., Figeys D., Andrews B. (2012). Exploring the yeast acetylome using functional genomics. Cell.

[53] Sandmeier J.J., French S., Osheim Y., Cheung W.L., Gallo C.M., Beyer A.L., Smith J.S. (2002). RPD3 is required for the inactivation of yeast ribosomal DNA genes in stationary phase. EMBO J..

[54] Diss G., Ascencio D., DeLuna A., Landry C.R. (2014). Molecular mechanisms of paralogous compensation and the robustness of cellular networks. J. Exp. Zool. B Mol. Dev. Evol..

[55] Costanzo M., Baryshnikova A., Bellay J., Kim Y., Spear E.D., Sevier C.S., Ding H., Koh J.L.Y., Toufighi K., Mostafavi S. (2010). The genetic landscape of a cell. Science.

[56] Segre D., Deluna A., Church G.M., Kishony R. (2005). Modular epistasis in yeast metabolism. Nat. Genet..

[57] Wolfe K.H., Shields D.C. (1997). Molecular evidence for an ancient duplication of the entire yeast genome. Nature.

[58] Iniguez L.P., Hernandez G. (2017). The Evolutionary Relationship between Alternative Splicing and Gene Duplication. Front. Genet..

[59] Grys B.T., Lo D.S., Sahin N., Kraus O.Z., Morris Q., Boone C., Andrews B.J. (2017). Machine learning and computer vision approaches for phenotypic profiling. J. Cell Biol..

[60] Kuzmin E., Sharifpoor S., Baryshnikova A., Costanzo M., Myers C.L., Andrews B.J., Boone C. (2014). Synthetic genetic array analysis for global mapping of genetic networks in yeast. Methods Mol. Biol..

[61] Kuzmin E., Costanzo M., Andrews B., Boone C. (2016). Synthetic Genetic Array Analysis. Cold Spring Harb. Protoc..

[62] Kuzmin E., Costanzo M., Andrews B., Boone C. (2016). Synthetic Genetic Arrays: Automation of Yeast Genetics. Cold Spring Harb. Protoc..

[63] Cox M.J., Chong Y.T., Boone C., Andrews B. (2016). Liquid Growth of Arrayed Fluorescently Tagged Saccharomyces cerevisiae Strains for Live-Cell High-Throughput Microscopy Screens. Cold Spring Harb. Protoc..

[64] Virtanen P., Gommers R., Oliphant T.E., Haberland M., Reddy T., Cournapeau D., Burovski E., Peterson P., Weckesser W., Bright J. (2020). SciPy 1.0: fundamental algorithms for scientific computing in Python. Nat. Methods.

[65] Ronneberger O., Fischer P., Brox T., Navab N., Hornegger J., Wells W.M., Frangi A.F. (2015). Net: Convolutional Networks for Biomedical Image Segmentation.

[66] Iakubovskii P. (2019).

[67] Litsios A., Grys B.T., Kraus O.Z., Friesen H., Ross C., Masinas M.P.D., Forster D.T., Couvillion M.T., Timmermann S., Billmann M. (2024). Proteome-scale movements and compartment connectivity during the eukaryotic cell cycle. Cell.

[68] He K.Z.,X., Ren S., Sun J. (2016). Identity Mappings in Deep Residual Networks. arXiv.

[69] Paszke A.G.,S., Massa F., Lerer A., Bradbury J., Chanan G., Killeen T., Lin Z., Gimelshein N., Antiga L., Desmaison A. (2019). NeurIPS Proceedings.

[70] He K., Zhang X., Ren S., Sun J. (2015). IEEE International Conference on Computer Vision (ICCV).

[71] Falcon W.B.,J., Wälchli A., Eggert N., Schock J., Jordan J., Skafte N., Bereznyuk V., Harris E., Murrell T., Yu P. (2020). PyTorchLightning/pytorch-lightning: 0.7.6 release. Zenodo.

[72] Kingma D.P.B.,J. (2014). Adam: A Method for Stochastic Optimization. arXiv.

[73] Loshchilov I.H.,F. (2016). SGDR: Stochastic Gradient Descent with Warm Restarts. arXiv.

[74] van der Walt S.J.S.,J.L., Nunez-Iglesias J., Boulogne F., Warner J.D., Yager N., Gouillart E., Yu T. (2014). the scikit-image contributors. 2014. scikit-image: image processing in Python. PeerJ.

[75] Buslaev A., Iglovikov V.I., Khvedchenya E., Parinov A., Druzhinin M., Kalinin A.A. (2020). Albumentations: Fast and Flexible Image Augmentations. Information.

[76] Oughtred R., Rust J., Chang C., Breitkreutz B.J., Stark C., Willems A., Boucher L., Leung G., Kolas N., Zhang F. (2021). The BioGRID database: A comprehensive biomedical resource of curated protein, genetic, and chemical interactions. Protein Sci..

[77] Krogan N.J., Cagney G., Yu H., Zhong G., Guo X., Ignatchenko A., Li J., Pu S., Datta N., Tikuisis A.P. (2006). Global landscape of protein complexes in the yeast Saccharomyces cerevisiae. Nature.

[78] Tarassov K., Messier V., Landry C.R., Radinovic S., Serna Molina M.M., Shames I., Malitskaya Y., Vogel J., Bussey H., Michnick S.W. (2008). An in vivo map of the yeast protein interactome. Science.

[79] Yu H., Braun P., Yildirim M.A., Lemmens I., Venkatesan K., Sahalie J., Hirozane-Kishikawa T., Gebreab F., Li N., Simonis N. (2008). High-quality binary protein interaction map of the yeast interactome network. Science.

[80] Babu M., Vlasblom J., Pu S., Guo X., Graham C., Bean B.D.M., Burston H.E., Vizeacoumar F.J., Snider J., Phanse S. (2012). Interaction landscape of membrane-protein complexes in Saccharomyces cerevisiae. Nature.

[81] Gavin A.C., Aloy P., Grandi P., Krause R., Boesche M., Marzioch M., Rau C., Jensen L.J., Bastuck S., Dümpelfeld B. (2006). Proteome survey reveals modularity of the yeast cell machinery. Nature.

[82] Hagberg A.S., D; Swart P. (2008). Proceedings of the 7th Python in Science Conference.

[83] Breker M., Gymrek M., Moldavski O., Schuldiner M. (2014). LoQAtE--Localization and Quantitation ATlas of the yeast proteomE. A new tool for multiparametric dissection of single-protein behavior in response to biological perturbations in yeast. Nucleic Acids Res..

[84] Thumuluri V., Almagro Armenteros J.J., Johansen A.R., Nielsen H., Winther O. (2022). DeepLoc 2.0: multi-label subcellular localization prediction using protein language models. Nucleic Acids Res..

[85] Sheff M.A., Thorn K.S. (2004). Optimized cassettes for fluorescent protein tagging in Saccharomyces cerevisiae. Yeast.

[86] Lu A.X., Zarin T., Hsu I.S., Moses A.M. (2019). YeastSpotter: accurate and parameter-free web segmentation for microscopy images of yeast cells. Bioinformatics.

[87] Dandage R. (2023). Htsimaging: High-Throughput Single-Cell Imaging Analysis in Python.

[88] Seabold S., Perktold J. (2010). Statsmodels: Econometric and Statistical Modeling with Python. SciPy Proc..

[89] Pedregosa F., Varoquaux G., Gramfort A., Michel V., Thirion B., Grisel O., Blondel M., Prettenhofer P., Weiss R., Dubourg V. (2011). Scikit-learn: Machine Learning in Python. J. Mach. Learn. Res..

[90] Harris C.R., Millman K.J., van der Walt S.J., Gommers R., Virtanen P., Cournapeau D., Wieser E., Taylor J., Berg S., Smith N.J. (2020). Array programming with NumPy. Nature.

[91] team T.p.d. (2023). Pandas-Dev/Pandas: Pandas.

[92] Hunter J.D. (2007). Matplotlib: A 2D Graphics Environment. Comput. Sci. Eng..

[93] Waskom M.L., Waskom M.L. (2021). Seaborn: statistical data visualization. J. Open Source Softw..

[94] Dandage R. (2023). Roux: Streamlined and Versatile Data Processing Toolkit.

