# Supplementary material for: Single-cell imaging of protein dynamics of paralogs reveals sources of gene retention
Source: iScience. 2025 May 27;28(7):112771. doi: 10.1016/j.isci.2025.112771 (PMC12205598; doi:10.1016/j.isci.2025.112771)
Supplement: Document S1. Figures S1–S8 [file mmc1.pdf]

iScience, Volume *28*

Supplemental information

Single-cell imaging of protein dynamics of paralogs reveals sources of gene retention

Rohan Dandage, Mikhail Papkov, Brittany M. Greco, Vanessa Pereira, Dmytro Fishman, Helena Friesen, Kyle Wang, Erin B. Styles, Oren Kraus, Benjamin Grys, Gerardo Zapata, Francois Lefebvre, Guillaume Bourque, Charles Boone, Brenda J. Andrews, Leopold Parts, and Elena Kuzmin

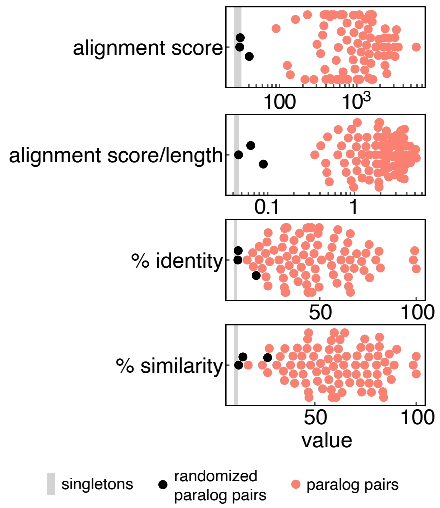

Figure S1. Sequence comparison of randomized paralog pairs with the paralog pairs examined in the study. The alignment metrics were obtained using the Needle algorithm, (EMBOSS:6.5.7.0). Paralog pairs examined in this study are shown in red, randomized paralog pairs are in black. For singletons, nonoverlapping 5 resampled subsets of 200 genes were randomly paired to produce 82 pairs for each subset. The metrics for each resampled set were averaged and then the confidence interval was determined by calculating mean+/-1*std across the subsets. The confidence interval is shown in gray.

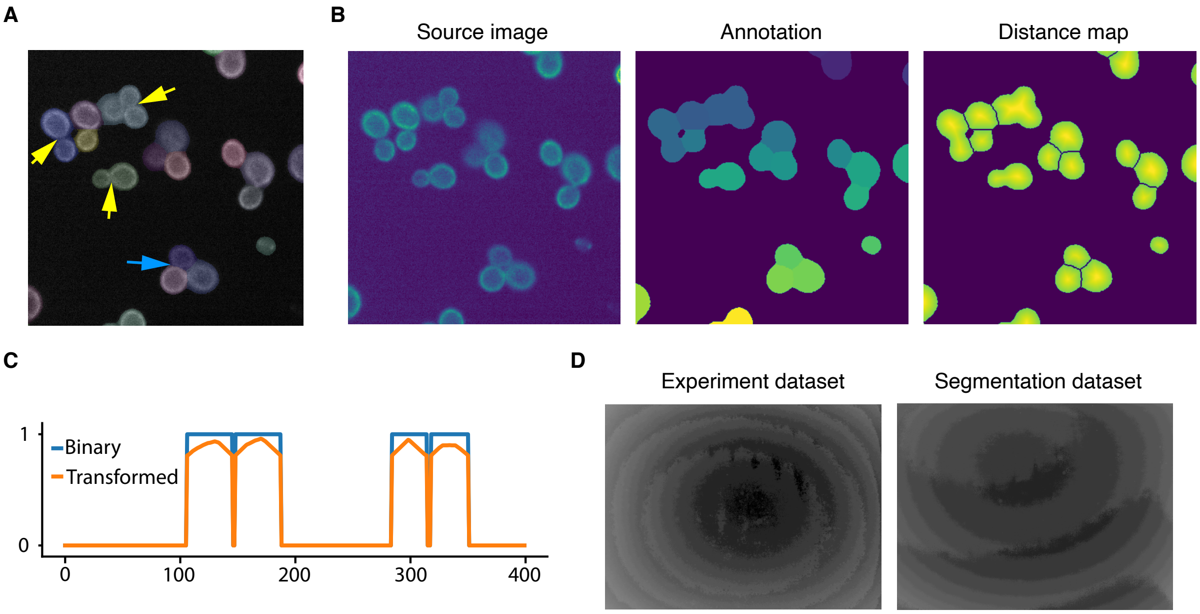

Figure S2. Optimization of the image segmentation. (A) A representative cropped microscopy image, with object annotation overlayed. The yeast cells in the ground truth are shown using yellow arrows. An example of fully separated cells is shown using the blue arrow. **(B)** Illustration of the proposed annotation processing into distance maps. Left: cropped microscopy image; center: ground truth object annotation; right: output distance map. **(C)** Vertical cross-sections of the binary segmentation and the distance map. **(D)** Log-transformed read-out noise highlights the uneven illumination pattern in both datasets.

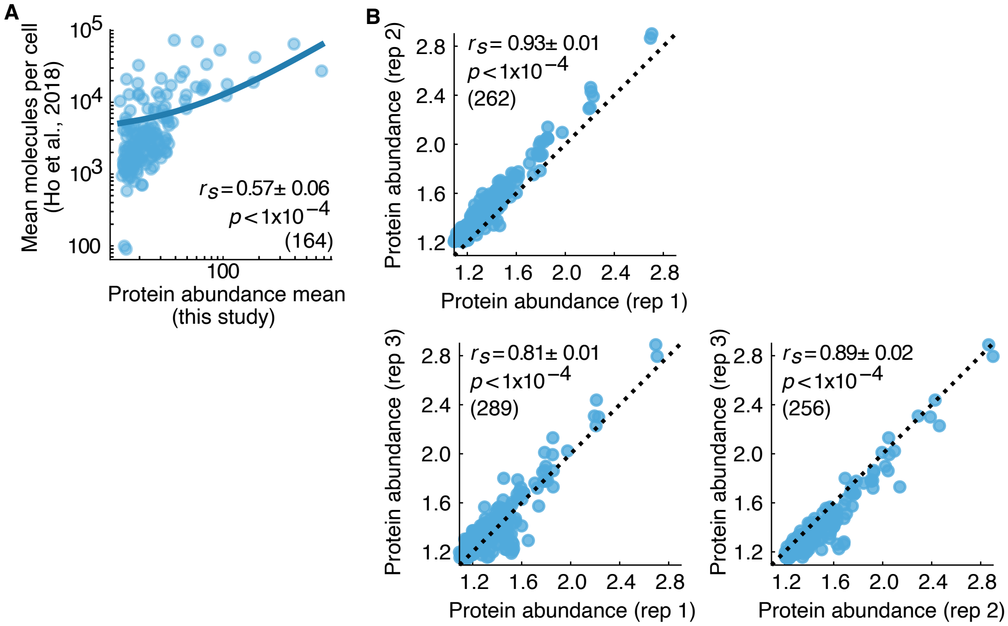

Figure S3. Protein abundance conformity with the known standard and reproducibility across replicate measurements. (A) Correlation of the abundance values in the wild-type background with the reference values (Ho et al 2018). The line indicates the fitted regression model of order 1. **(B)** Correlations of the protein abundance values (log_{10}-scaled) in the wild-type and deletion backgrounds across replicates (rep). *rs:* Spearman's rank correlation coefficient; *p*: p-value associated with the correlation. The robustness of the correlations was tested by five rounds of resampling.

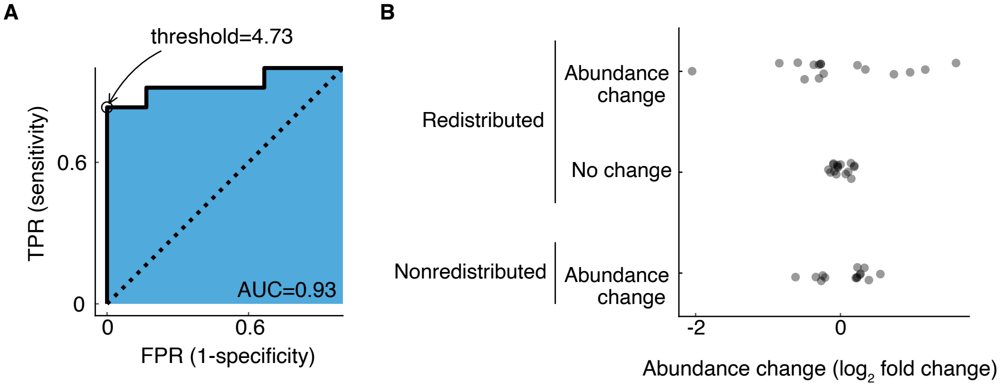

Figure S4. The classification of redistributed paralogs and comparison with the relative abundance change. (A) Receiver Operating Characteristic (ROC) curve for the classification of the redistributed paralogs. **(B)** Relative abundance change scores of paralogs stratified by the significance of redistribution and abundance change.

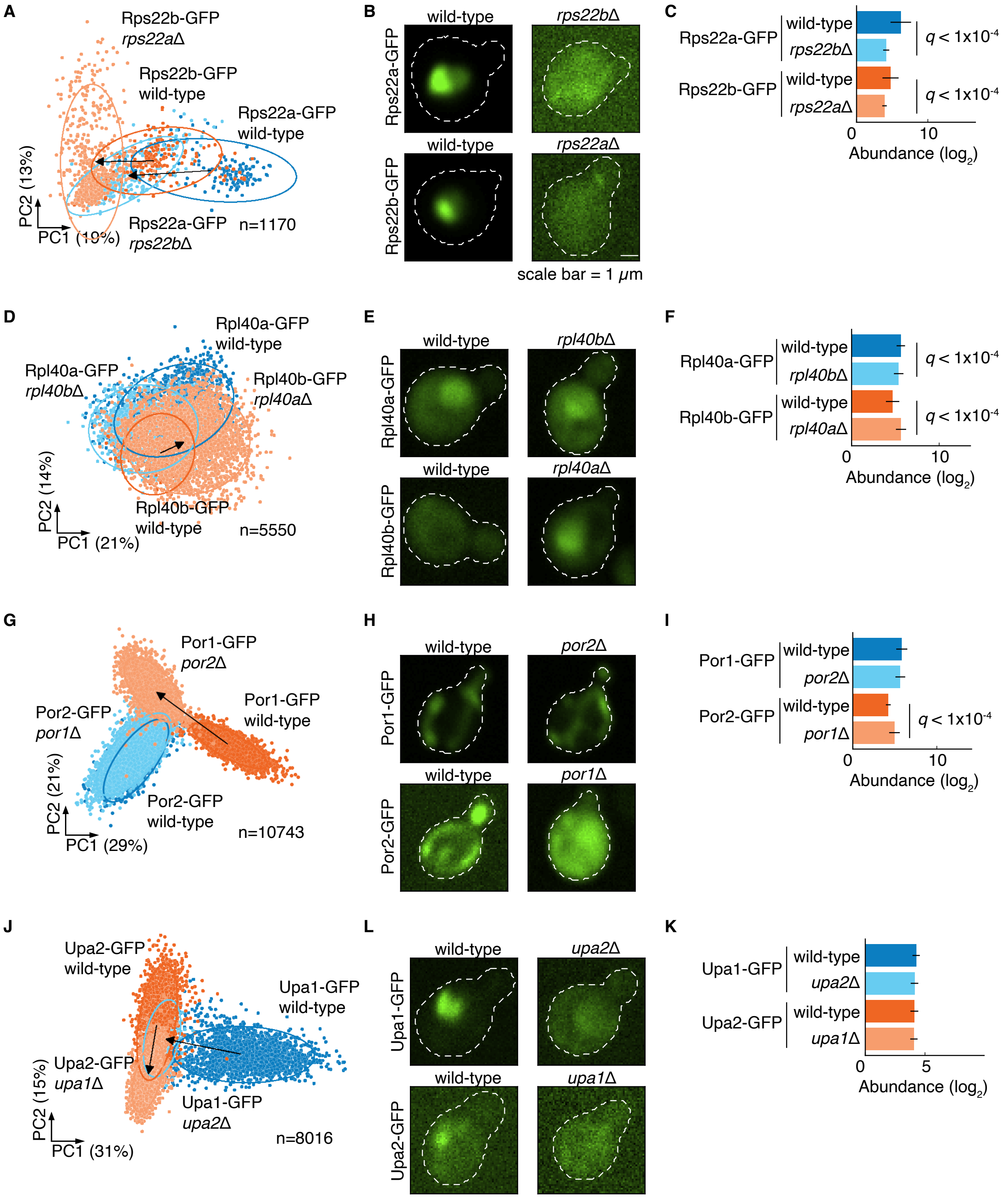

Figure S5. Redistribution, relative abundance and relocalization of paralog pairs. Redistribution of the **(A)** Rps22a-Rps22b, **(D)** Rpl40a-Rpl40b, **(G)** Por1-Por2 and **(J)** Upa1-Upa2 pairs, are represented on a dimensionality reduced Principal component analysis (PCA) space of z-score normalized features for each construct. Redistributed paralogous proteins are depicted by the arrow connecting the centroids of the clusters corresponding to the wild-type (dark blue/orange) and deletion (light blue/orange) backgrounds of the paralog. The percentage variances explained by the PCs are indicated in parentheses. The PCA plot is used for the visual representation of the redistribution only. **(B, E, H, L)** Micrographs of representative yeast cells of respective paralog pairs. **(C, F, I, K)** Relative abundance changes of the respective pairs. The error bars show the 95% confidence intervals of the means. q: FDR-corrected p-value. Wild-type (dark blue/orange) and deletion (light blue/orange) backgrounds of the paralog.

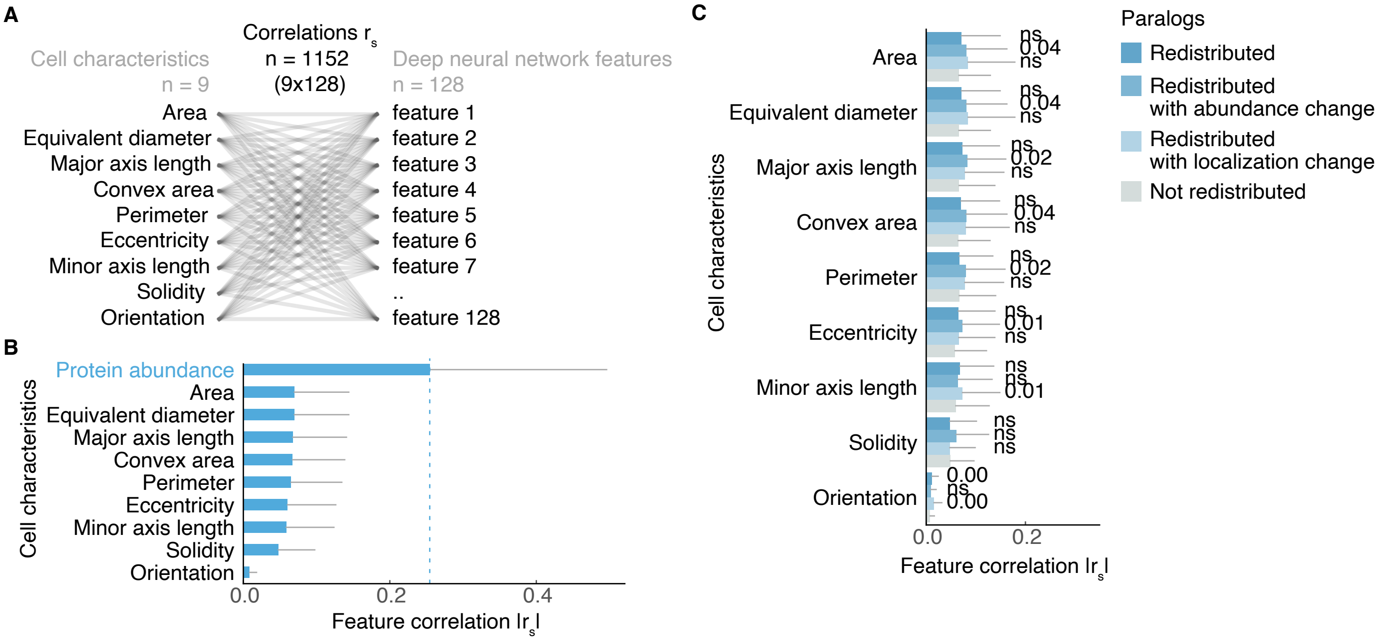

Figure S6. Correlations of the neural network derived features with morphological characteristics. (A) Schematic showing the method used in the correlation analysis. Cell characteristics related to cellular morphology including size and shape were correlated with protein dynamics features obtained from the neural network. **(B)** The correlation coefficients between neural network derived features and morphological characteristics for all the paralogs are shown. The correlation with cellular protein abundance is shown for reference. Its third quartile value (Q3) is indicated by the dotted line. **(C)** The comparison of cell features correlation coefficients for paralogs belonging to the categories indicated in the legend. |rs|: Absolute value of the rank correlation coefficients calculated by (scale-agnostic) Spearman method (see Methods). In panels B and C, the extent of the bars is up to the third quartile (Q3), and the lines extend to Q3+1.5*IQR. Statistical significance was determined using a two-sided Mann-Whitney U-test. ns: non-significant.

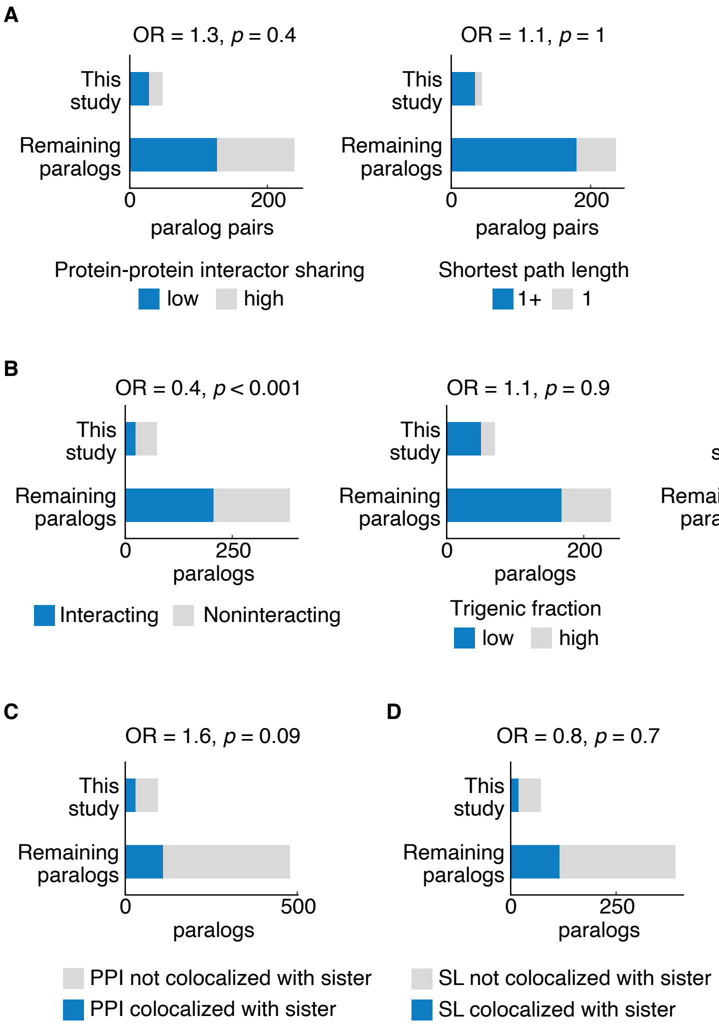

Figure S7. Comparison of paralog features between the paralog pairs used in this study and the remaining WGD paralogs. (A) Protein-protein interaction (PPI) network features, used in Figure 6A. **(B)** Genetic interaction network features, used in Figure 6B. **(C)** Colocalization of the PPI and genetic interaction interactors of the paralogs, used in Figure 6C. OR: Odds ratio determined by Fisher's exact test, *p*: p-value associated with the OR.

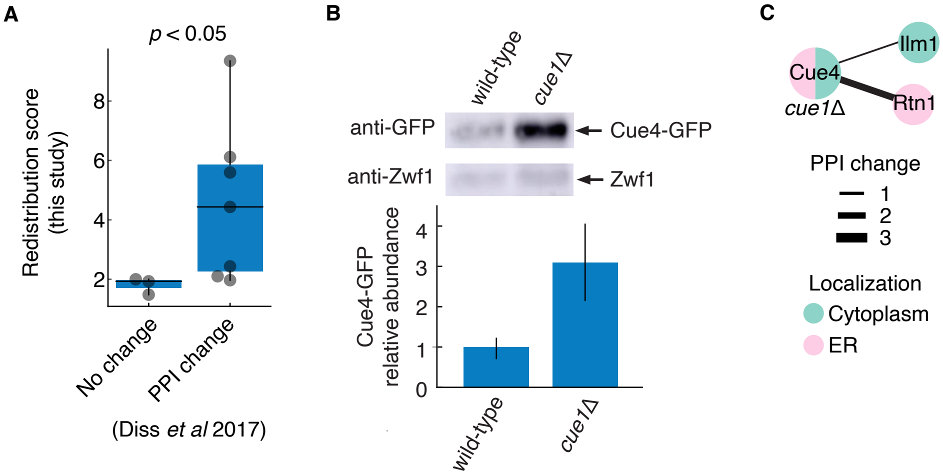

Figure S8. Redistributed paralogs show change in protein-protein interactions (PPIs). (A) The comparison of the redistribution scores (this study) for proteins that show no change in PPIs with those that show at least one PPI change when their paralog is deleted. Statistical significance was determined using a two-sided Mann-Whitney U-test. **(B)** Western blot using whole-cell lysate shows increased mean protein expression of Cue4p in *cue1*Δ compared to wild-type background. Quantification is normalized to the loading control Zwf1p, error bars denote STD, n = 3. **(C)** Cue1-Cue4 represent an example of paralogous proteins in which only Cue4 shows redistribution (this study) and a corresponding PPI change upon *CUE1* deletion. The colors of the nodes indicate the subcellular compartments. Edges connect interacting proteins; edge thickness shows PPI change. PPI change was obtained from a previous study (Diss et al. 2017). In the boxplots, the central line indicates the median, the extent of the box is from the first quartile (Q1) to the third quartile (Q3), and the whiskers extend to Q1-1.5*IQR and Q3+1.5*IQR.
